# Associations between air pollution and multimorbidity in the UK Biobank: A cross-sectional study

**DOI:** 10.3389/fpubh.2022.1035415

**Published:** 2022-12-02

**Authors:** Amy Ronaldson, Jorge Arias de la Torre, Mark Ashworth, Anna L. Hansell, Matthew Hotopf, Ian Mudway, Rob Stewart, Alex Dregan, Ioannis Bakolis

**Affiliations:** ^1^Centre for Implementation Science, Health Service and Population Research Department, Institute of Psychiatry, Psychology and Neuroscience (IoPPN), King's College London, London, United Kingdom; ^2^CIBER Epidemiology and Public Health (CIBERESP), Madrid, Spain; ^3^Institute of Biomedicine (IBIOMED), University of Leon, Leon, Spain; ^4^School of Life Course and Population Sciences, King's College London, London, United Kingdom; ^5^Centre for Environmental Health and Sustainability, University of Leicester, Leicester, United Kingdom; ^6^National Institute for Health and Care Research, Health Protection Research Unit (HPRU) in Environmental Exposures and Health at the University of Leicester, Leicester, United Kingdom; ^7^Department of Psychological Medicine, King's College London, IoPPN and South London and Maudsley NHS Foundation Trust, London, United Kingdom; ^8^South London and Maudsley NHS Foundation Trust, London, United Kingdom; ^9^National Institute for Health and Care Research, Health Protection Unit in Environmental Exposures and Health, Imperial College London, London, United Kingdom; ^10^MRC Centre for Environment and Health, School of Public Health, Faculty of Medicine, Imperial College London, London, United Kingdom; ^11^Department of Biostatistics and Health Informatics, IoPPN, King's College London, London, United Kingdom

**Keywords:** air pollution, particulate matter, nitrogen dioxide, multimorbidity, health status, exploratory factor analysis

## Abstract

**Background:**

Long-term exposure to air pollution concentrations is known to be adversely associated with a broad range of single non-communicable diseases, but its role in multimorbidity has not been investigated in the UK. We aimed to assess associations between long-term air pollution exposure and multimorbidity status, severity, and patterns using the UK Biobank cohort.

**Methods:**

Multimorbidity status was calculated based on 41 physical and mental conditions. We assessed cross-sectional associations between annual modeled particulate matter (PM)_2.5_, PM_coarse_, PM_10_, and nitrogen dioxide (NO_2_) concentrations (μg/m^3^–modeled to residential address) and multimorbidity status at the baseline assessment (2006–2010) in 364,144 people (mean age: 52.2 ± 8.1 years, 52.6% female). Air pollutants were categorized into quartiles to assess dose-response associations. Among those with multimorbidity (≥2 conditions; *n* = 156,395) we assessed associations between air pollutant exposure levels and multimorbidity severity and multimorbidity patterns, which were identified using exploratory factor analysis. Associations were explored using generalized linear models adjusted for sociodemographic, behavioral, and environmental indicators.

**Results:**

Higher exposures to PM_2.5_, and NO_2_ were associated with multimorbidity status in a dose-dependent manner. These associations were strongest when we compared the highest air pollution quartile (quartile 4: Q4) with the lowest quartile (Q1) [PM_2.5_: adjusted odds ratio (adjOR) = 1.21 (95% CI = 1.18, 1.24); NO_2_: adjOR = 1.19 (95 % CI = 1.16, 1.23)]. We also observed dose-response associations between air pollutant exposures and multimorbidity severity scores. We identified 11 multimorbidity patterns. Air pollution was associated with several multimorbidity patterns with strongest associations (Q4 vs. Q1) observed for neurological (stroke, epilepsy, alcohol/substance dependency) [PM_2.5_: adjOR = 1.31 (95% CI = 1.14, 1.51); NO_2_: adjOR = 1.33 (95% CI = 1.11, 1.60)] and respiratory patterns (COPD, asthma) [PM_2.5_: adjOR = 1.24 (95% CI = 1.16, 1.33); NO_2_: adjOR = 1.26 (95% CI = 1.15, 1.38)].

**Conclusions:**

This cross-sectional study provides evidence that exposure to air pollution might be associated with having multimorbid, multi-organ conditions. Longitudinal studies are needed to further explore these associations.

## Introduction

Outdoor air pollution is the leading environmental cause of premature mortality globally, contributing to between four and nine million deaths annually (1). Short- and long-term exposure to air pollution is associated with the development and exacerbation of both acute and chronic diseases, including cardiovascular disease (2), stroke (3), respiratory disease (4) [e.g., asthma (5), chronic obstructive pulmonary disorder (COPD) (6)], diabetes (7), neurodegenerative conditions (8), as well as common (9, 10) and severe mental health conditions (11, 12). Following the recent World Health Organization (WHO) revision of the annual PM_2.5_ guideline value to 5 μg/m^3^ and NO_2_ guideline value to 10μg/m^3^, it is now estimated that over 99% of the world population is exposed to unhealthy levels of pollutants, further reinforcing air pollution as one of the main public health priorities worldwide (13).

Since air pollution is likely to affect several body systems simultaneously, it is plausible that exposure to air pollutants might result in the accumulation of multiple long-term conditions (LTCs), leading to multimorbidity. Multimorbidity—defined as the co-existence of two or more LTCs—is increasingly common, affecting ~27% of adults in UK primary care services (14) and this is set to increase significantly in the future (15). To our knowledge, only one study has examined the role of air pollution in multimorbidity. Hu et al. found that both lower and higher levels of air pollution were associated with the accumulation of diseases (16). They identified three multimorbidity patterns and found that air pollution was associated with these patterns to varying degrees. As this study was carried out in China, where both ambient and household levels of air pollution are particularly high (17), it is not clear how generalisable the results are to the UK. Moreover, this study only assessed effects of PM_2.5_ and used a small number of LTCs to define multimorbidity. Therefore, in the current study, we employed more comprehensive measures of both air pollution and multimorbidity to assess associations in the UK.

The aim of the current study was to examine associations between long-term exposure to several modeled residential air pollutants with both multimorbidity status and severity in a large cohort of adults from the UK Biobank. The evidence from China suggests that exposure to air pollution might increase vulnerability to certain patterns of conditions more than others; therefore, we also identified specific patterns of multimorbidity and examined the extent to which exposure to specific pollutants associated with these patterns. Our primary hypothesis was that higher air pollution exposures would be associated with increased likelihood of multimorbidity, as well as more severe multimorbidity in those affected. We also hypothesized that different air pollutants would associate selectively with different multimorbidity patterns.

## Methods

### Study design and population

In this cross-sectional, observational study we analyzed data from the UK Biobank, a large population-based study designed to investigate the development of disease in UK adults of middle- and older-age. Between 2006 and 2010, over 500,000 people aged 40–69 years attended a baseline assessment across 22 centers in England, Scotland, and Wales (18). Participants had to live within 25 miles of an assessment center and had to be registered with a general practitioner (GP) to enroll in the UK Biobank. At the baseline assessment, sociodemographic, lifestyle, and medical information were gathered. In a computer assisted personal interview, trained interviewers gathered patients self-reported information about medical diagnoses. Further information about diagnoses were also gathered through linkages with hospital records from the Hospital Episodes Statistics (HES) database. All participants gave informed consent. Ethical approval for the UK Biobank was granted by the NHS National Research Ethics Service (16/NW/0274).

The current study included participants with complete data for the exposure variables of interest to ensure that any differences between models were not due to selection bias (*n* = 364,144, see Supplementary Figure 1 for a flowchart depicting sample selection). As a result, the study sample comprised participants from England only as some covariates (residential greenspace, noise pollution) were not collected in Wales or Scotland.

### Measures

#### Exposure: Air pollution estimates

Air pollution estimates for 2010 were derived using a Land Use Regression (LUR) model developed as part of the European Study of Cohorts of Air Pollution Effects (ESCAPE) (http://www.escapeproject.eu/). Estimates were modeled to the x-y coordinate of the residential addresses of each UK biobank participant at baseline. Exposure to fine particulate matter (PM) with a diameter <2.5 μm (PM_2.5_), coarse PM with a diameter between 2.5 and 10 μm (PM_coarse_), PM with a 50% cut-off aerodynamic diameter of 10 μm (PM_10_), and nitrogen dioxide (NO_2_), were modeled as annual average values in μg/m^3^. Detailed descriptions of the models and their evaluation has been provided previously (19, 20).

#### Outcome: Multimorbidity status

Multimorbidity status was determined using self-reported medical diagnoses from the baseline assessment and primary and secondary diagnoses from linked HES data (ICD-10 codes). The multimorbidity measure was based on previous work with the UK Biobank where a total of 36 physical (e.g., diabetes, coronary heart disease, cancer) and five mental health LTCs (e.g., depression, schizophrenia/bipolar disorder) were classified (see Supplementary Table 1 for a full list of conditions) (21). Note that depression and anxiety were determined using multiple sources: self-report, linked HES data, as well as scores on the Patient Health Questionnaire (PHQ)-4. Physical/mental multimorbidity status was determined using a cut-off of two or more co-existing LTCs. We created an ordinal multimorbidity status variable by grouping participants into; 0 or 1 condition (no multimorbidity); 2 conditions; 3 conditions; 4 or more conditions in order to assess potential dose-response associations with air pollution exposure.

#### Outcome: Multimorbidity severity

Amongst participants with multimorbidity (≥2 LTCs), we generated a multimorbidity severity score using severity weights from the Cambridge Multimorbidity Score (CMS) (22). The CMS generated severity weights based on primary care consultations, unplanned hospital admissions, and mortality for a list of 37 LTCs. Thirty-three of these LTCs were included in the current study. Severity scores were not available for 8 of the LTCs in the current study. Therefore, multiple imputation using chained equations (23) was performed to generate severity scores for these 8 LTCs. The CMS severity scores, mean age and proportion of female participants for each LTC were included in the multiple imputation, as well as whether a LTC was considered to render a participant “healthy” or “unhealthy” according to a classification developed by the Reinsurance Group of America (24). Severity weights for each condition are presented in Supplementary Table 2. An overall multimorbidity severity score was calculated by summing these weights for each participant. These scores were further categorized into quartiles due to skewness of the data.

#### Outcome: Multimorbidity patterns

Among participants with multimorbidity, we used exploratory factor analysis (EFA) to identify patterns of multimorbidity from the 36 physical and 5 mental conditions measured at the baseline assessment. This method assumes that LTCs share common underlying etiologies and groups them accordingly. As per previous multimorbidity research, we excluded LTCs with a prevalence of <1% from the EFA in order to create more refined multimorbidity patterns (25). Since LTCs were coded as binary variables (yes/no), we applied the principal factor method based on a tetrachoric correlation matrix. Bartlett's Test of Sphericity and the Kaiser-Meyer-Olkin (KMO) measure of sampling adequacy were used to assess the suitability of the data for EFA. We determined the appropriate factor solution using Eigenvalues (>1), the shape of the screeplot, and parallel analysis. Oblique rotation was applied and each resulting factor loading represented the strength of the association between the LTC and the multimorbidity pattern (i.e., latent factor). We assigned a condition to a specific multimorbidity pattern if the rotated factor loading was at least +/−0.4 (21).

People with multimorbidity were assigned to specific multimorbidity patterns if they had at least two of the LTCs included in the pattern. For each multimorbidity pattern we created a binary variable where “0 = no multimorbidity” and “1 = assigned to multimorbidity pattern”.

#### Covariates

Several sociodemographic, behavioral, and environmental covariates known to associate with multimorbidity and/or air pollution were included. Individual-level sociodemographic factors such as age, gender, ethnicity, education level, employment status, and household income are associated with multimorbidity (26, 27) and were therefore included as covariates. Alcohol intake frequency, smoking status, physical activity, and body mass index (BMI) are associated with physical health status (28, 29) so were included as covariates. The UK Biobank also provides estimates of other relevant measures of the local environment which were included as covariates: distance of residence to nearest major road, traffic intensity on nearest major road, 24-h noise pollution, and residential greenspace (within 1,000 m of residential address). More detail on covariates is provided in the Supplementary Methods section.

#### Statistical analysis

We summarized sample characteristics using means and standard deviations, medians and interquartile ranges (IQR), and frequencies for the overall sample, and for those with and without multimorbidity. Differences between those with and without multimorbidity were examined using independent *t*-tests and χ^2^ tests. Moreover, we used these tests to investigate whether there were differences between the analytical sample and those excluded from the study due to missing data.

Air pollutant variables were rescaled to IQR increments to allow the calculation of effect estimates for comparable increases across pollutants, which have differences in concentration ranges. Air pollutants were also categorized into quartiles to permit assessment of dose-response associations.

We examined associations between each air pollutant (as both continuous values and quartiles) and multimorbidity status (no multimorbidity, 2 conditions, 3 conditions, 4 or more conditions) using ordinal regression models. Among people with multimorbidity, multimorbidity severity scores were positively skewed and were therefore recategorized into quartiles. We then examined associations between each air pollutant and multimorbidity severity quartiles using ordinal regression models. Following identification of multimorbidity patterns using exploratory factor analysis as described earlier in the Methods section, we examined associations between each air pollutant (continuous values) and multimorbidity patterns using logistic regression models.

All models were fitted separately for each outcome and each pollutant with increasing degrees of covariate adjustment in order to assess the potential impact of different covariate types. In Model 1 we adjusted for age and sex. Individual-level sociodemographic factors (ethnicity, education level, employment status, household income) were introduced in Model 2. Model 3 included health behaviors (alcohol intake frequency, smoking status, physical activity, BMI). Factors relating to the surrounding environment were added to Model 4 (proximity of the nearest major road and traffic intensity on that road, 24 h noise pollution, and residential greenspace).

As recommended for observational research (30), we calculated E-values. E-values represent the minimum strength of association that an unmeasured confounder would need to have with both air pollution and multimorbidity status in order to explain away the significant effects reported in this study.

Data analyses were conducted in STATA 17.0 (Stata Corp LLP, College Station, TX).

#### Sensitivity analyses

Three sensitivity analyses were performed:

To address misclassification and uncertainties over diagnosis and exposure we restricted our analyses to those who completed the baseline assessment in 2010, so that exposure and outcome were measured in the same year.

To account for possible clustering of individuals at assessment centers, we examined associations using mixed-effects ordered logistic regression where exposure to air pollution was considered at level one (fixed effects) and assessment center was considered at level 2 (random effects).

We ran two-pollutant models where each pollutant was included as a covariate with every other pollutant in order to examine co-pollutant confounding.

#### Patient and public involvement

Details of patient and public involvement in the UK Biobank are available online (https://www.ukbiobank.ac.uk/learn-more-about-uk-biobank). Study participants were not specifically involved in the design and implementation of this study.

## Results

### Sample characteristics

Sample characteristics are presented in Table 1 for the overall sample (*N* = 364,144) and for those with multimorbidity (*N* = 156,395, 42.9%) and those without (*N* = 207,749, 57.1%). The rate of multimorbidity is higher in the current study than reported in previous UK Biobank studies (14). This is due to the inclusion of the PHQ-4 in the measurement of depression and anxiety which results in a more sensitive measure of these conditions (21). Participants with multimorbidity were older, more likely to be female, and more likely to be from an ethnic minority group. Moreover, they were less likely to have a higher level of education, less likely to be employed, and more likely to earn <£18,000 per year compared to those with none or one condition. In terms of health behaviors, those with multimorbidity were less likely to drink alcohol regularly and were more likely to be past or current smokers. Physical activity was lower in those with multimorbidity, and obesity levels were higher.

**Table 1:** Sample characteristics.

	**Overall sample** ** (*N* = 364,144)**	**Multimorbidity ** **(*N* = 156,395, 42.9%)**	**No multimorbidity ** ** (*N* = 207,749, 57.1%)**	
	**M±SD or N(%)**	**M±SD or N(%)**	**M±SD or N(%)**	**P-value**
Age	56.24 ± 8.08	57.93 ± 7.80	54.97 ± 8.06	<0.001
Female	191,563 (52.6)	84,216 (53.8)	107,347 (51.7)	<0.001
Ethnicity				<0.001
White	346,026 (95.0)	147,530 (94.3)	198,496 (95.6)	
Asian/Asian British	6,362 (1.8)	3,432 (2.2)	2,930 (1.4)	
Black/Black British	5,607 (1.5)	2,710 (1.7)	2,897 (1.4)	
Mixed	2,147 (0.6)	891 (0.6)	1,256 (0.6)	
Other	4,002 (1.1)	1,832 (1.2)	2,170 (1.0)	
Education level				<0.001
High	110,330 (30.3)	44,269 (28.3)	66,061 (31.8)	
Intermediate	81,231 (22.3)	34,321 (21.9)	46,910 (22.6)	
Low	172,583 (47.4)	77,805 (49.8)	94,778 (45.6)	
Employment status				<0.001
Employed	221,091 (60.7)	78,556 (50.2)	142,535 (68.6)	
Retired	115,445 (31.7)	61,189 (39.1)	54,256 (26.1)	
Unemployed/volunteer/career	27,608 (7.6)	16,650 (10.6)	10,958 (5.3)	
Household income				<0.001
<£18,000	79,952 (22.0)	48,013 (30.7)	31,939 (15.4)	
£18,000 to £29,999	92,318 (23.4)	43,103 (27.6)	49,215 (23.7)	
£30,000 to £51,999	95,415 (26.2)	36,569 (23.4)	58,846 (28.3)	
£52,000 to £100,000	75,670 (20.8)	23,380 (15.0)	52,290 (25.2)	
>£100,000	20,789 (5.7)	5,330 (3.4)	15,459 (7.4)	
Alcohol intake frequency				<0.001
Daily/almost daily	77,939 (21.4)	31,241 (20.0)	46,698 (22.5)	
3–4 times per week	86,857 (23.9)	32,235 (20.6)	54,622 (26.3)	
1–2 times per week	92,935 (25.5)	37,762 (24.1)	55,173 (26.5)	
1–3 times per month	40,453 (11.1)	18,319 (11.7)	22,134 (10.7)	
Never/special occasions	65,960 (18.1)	36,838 (23.6)	29,122 (14.0)	
Current or past smoker	165,916 (45.6)	78,166 (50.0)	87,750 (42.2)	<0.001
Physical activity				<0.001
None	5,952 (1.6)	4,145 (2.6)	1,807 (0.9)	
Low	67,868 (18.6)	33,644 (21.5)	34,224 (16.4)	
Moderate	148,427 (40.8)	63,894 (40.9)	84,533 (40.7)	
Vigorous	141,897 (39.0)	54,712 (35.0)	87,185 (42.0)	
BMI (kg/m^2^)				<0.001
Underweight (<18.5)	1,835 (0.5)	751 (0.5)	1,084 (0.5)	
Normal weight (18.5–24.9)	119,812 (32.9)	40,464 (25.9)	79,348 (38.2)	
Overweight (25-29.9)	155,518 (42.7)	65,424 (41.8)	90,094 (43.4)	
Obesity (≥30)	86,979 (23.9)	49,756 (31.8)	37,223 (17.9)	
Nearest major road (1/m)	0.006 ± 0.016	0.006 ± 0.017	0.006 ± 0.015	0.007
Traffic intensity on nearest major road (vehicles/day)	23,194.3 ± 20,908.2	23,407.9 ± 20,998.1	23,033.5 ± 20,838.8	<0.001
Noise pollution 24 h (dB)	56.04 ± 4.27	56.05 ± 4.29	56.03 ± 4.24	0.096
Residential greenspace 1,000 m (%)	45.28 ± 21.64	44.90 ± 21.11	45.57 ± 22.02	<0.001
PM_2.5_ (μg/m^3^)	9.98 ± 1.05	10.03 ± 1.06	9.94 ± 1.04	<0.001
PM_10_ (μg/m^3^)	16.20 ± 1.88	16.25 ± 1.87	16.17 ± 1.88	<0.001
PM_coarse_ (μg/m^3^)	6.41 ± 0.89	6.42 ± 0.89	6.40 ± 0.88	<0.001
NO_2_ (μg/m^3^)	26.60 ± 7.61	26.87 ± 7.54	26.39 ± 7.65	<0.001
Number of conditions				<0.001
0 or 1 condition (no MM)	207,749 (57.1)	–	207,749 (100.0)	
2 conditions	75,689 (20.8)	75,689 (48.4)	–	
3 conditions	42,611 (11.7)	42,611 (27.2)	–	
4 or more conditions	38,095 (10.5)	38,095 (24.4)	–	
Multimorbidity severity score	0.71 ± 0.84	1.35 ± 0.86	0.23 ± 0.37	<0.001

BMI, body mass index; dB, decibel; MM, multimorbidity; NO, nitrogen oxide; PM, particulate matter.

Modeled estimates of residential exposure to all air pollutants were higher in those with multimorbidity. Proximity to nearest major road did not differ between those with and without multimorbidity, but the traffic intensity on that road was higher in people with multiple LTCs. Participants with multimorbidity had less access to residential greenspace, but there was no difference in 24-h noise pollution levels between those with and without multimorbidity.

Correlations between air pollutants are presented in Supplementary Table 3.

We investigated differences between the analytical sample (*N* = 364,144) and those excluded from the study on the basis of missing data (*N* = 138,455) (Supplementary Table 4). Excluded participants differed from the analytical sample on many sociodemographic factors, had higher levels of multimorbidity, and were exposed to higher levels of all air pollutants.

### Air pollution and multimorbidity status

**Figure 1:**
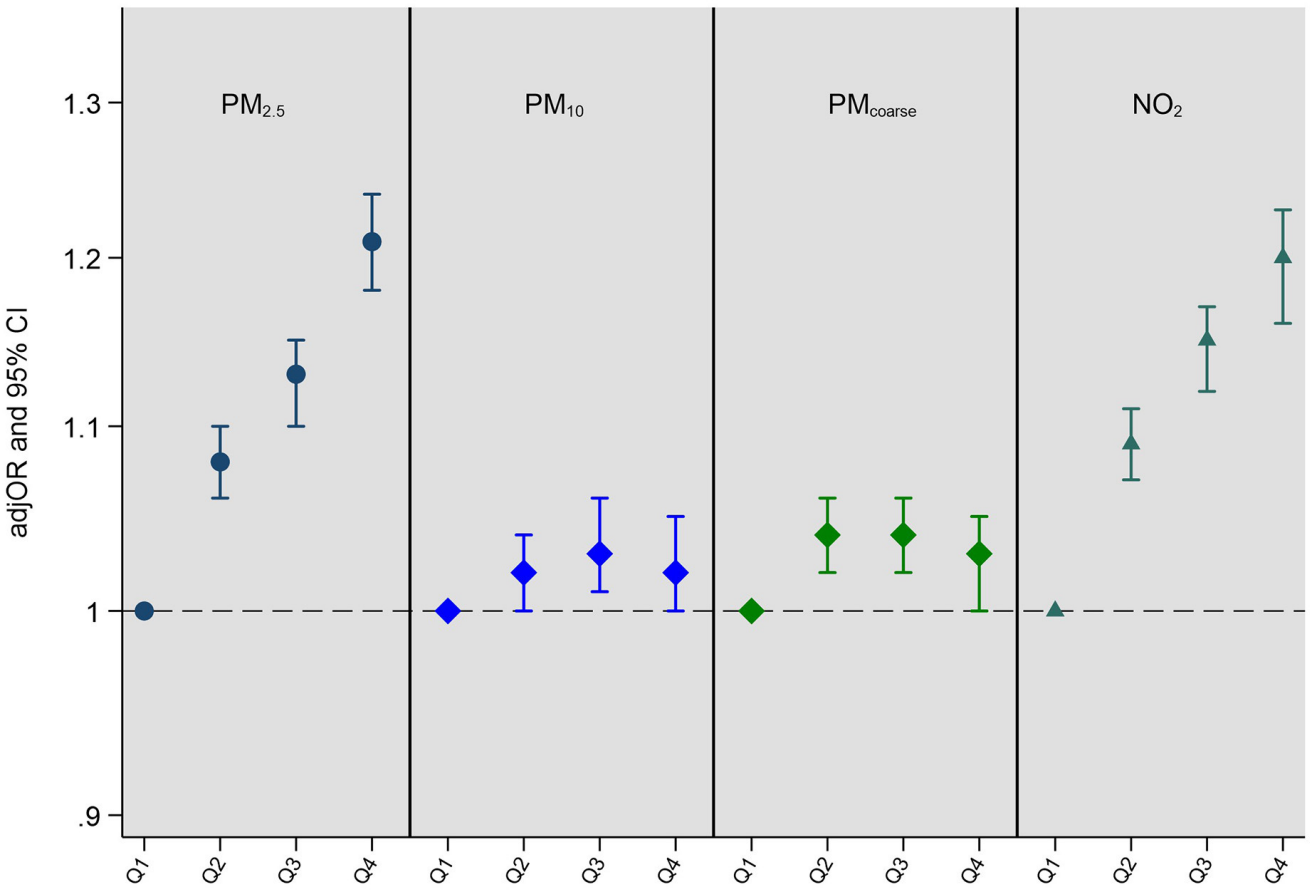
Adjusted odds ratios (adjOR) and their corresponding 95% confidence intervals (CI) from ordinal regressions looking at associations between exposure to air pollution (PM_2.5_, PM_10_, PM_coarse_, and NO_2_) and multimorbidity status (*N* = 364,144). Air pollutants (μg/m^3^) are categorized as quartiles in order to assess dose response associations, with the lowest quartile (Q1) acting as the reference quartile. All models are adjusted for age, sex, ethnicity, education level, employment status, household income, smoking status, alcohol intake frequency, BMI, physical activity, nearest major road, traffic intensity on nearest major road, residential green space (1,000 m) and 24 h noise pollution (Lden). The quartile cut-offs for NO_2_ were: Q1 <21.32 μg/m^3^, Q2 <26.09 μg/m^3^, Q3 <31.22 μg/m^3^ and Q4 ≥31.22 μg/m^3^; for PM_2.5_ they were: Q1 <9.29 μg/m^3^, Q2 <9.93 μg/m^3^, Q3 <10.56 μg/m^3^ and Q4 ≥10.56 μg/m^3^; for PM_coarse_ they were: Q1 <5.85 μg/m^3^, Q2 <6.11 μg/m^3^, Q3 <6.63 μg/m^3^ and Q4 ≥6.63 μg/m^3^; and for PM_10_ they were: Q1 <15.24 μg/m^3^, Q2 <16.03 μg/m^3^, Q3 <16.99 μg/m^3^ and Q4 ≥16.99 μg/m^3^. *P* values for trend for each air pollutant are as follows: PM_2.5_: *p* < 0.001; PM_10_: *p* = 0.002; PM_coarse_: *p* = 0.300; NO_2_: *p* < 0.001.

Fully adjusted associations (Model 4) between single air pollutant exposures and multimorbidity status are presented in Figure 1 (all models are presented in Supplementary Table 5). Greater exposure to PM_2.5_, and NO_2_ was associated with higher rates of multimorbidity. After full covariate adjustment (Model 4), clear dose-response associations emerged between air pollution quartiles and multimorbidity with the most pronounced associations seen when we compared the highest quartile with the lowest for PM_2.5_ (fully adjusted odds ratio (adjOR) = 1.21, 95% CI = 1.18–1.24); and NO_2_ (adjOR = 1.20, 95% CI = 1.16–1.23). There was no clear exposure response for PM_coarse_ or PM_10_. *P* values for trend for each air pollutant are as follows: PM_2.5_: *p* < 0.001; PM_10_: *p* = 0.002; PM_coarse_: *p* = 0.300; NO_2_: *p* < 0.001. Although the *p* value for trend for PM_10_ exposure is significant, the pattern of associations are not clear cut and appear to be driven by comparisons between the quartile 1 (lowest) and quartile 3 (second-highest) (see Supplementary Table 5).

E-values indicated that unmeasured confounding was unlikely to explain away a substantial proportion of the observed associations for PM_2.5_ and NO_2_ (Supplementary Tables 6A,B).

### Air pollution and multimorbidity severity

Multimorbidity severity scores ranged from −0.97 to 11.84. As shown in Table 2, when multimorbidity severity scores were converted to quartiles, dose-response associations were evident for PM_2.5_ and NO_2_. These associations were strongest for the highest quartiles (vs. the first quartile) of PM_2.5_ (adjOR = 1.11, 95% CI, 1.06–1.15), and NO_2_ (adjOR = 1.08, 95% CI, 1.04–1.13). PM_coarse_ and PM_10_ were not associated with multimorbidity severity.

**Table 2:** Associations between IQR increments in air pollution (*italics*), air pollution quartiles and multimorbidity severity scores in people with multimorbidity (*N* = 156,395).

		**Model 1**		**Model 2**		**Model 3**		**Model 4**	
	**M±SD**	**OR (95% CI)**	***p* value**	**OR (95% CI)**	***p* value**	**OR (95% CI)**	***p* value**	**OR (95% CI)**	***p* value**
PM_2.5_									
1	0.66 ± 0.80	Ref		Ref		Ref		Ref	
2	0.70 ± 0.83	1.07 (1.04 to 1.10)	<0.001	1.03 (1.00 to 1.06)	0.050	1.02 (0.99 to 1.05)	0.229	1.02 (0.99 to 1.05)	0.171
3	0.73 ± 0.85	1.15 (1.12 to 1.18)	<0.001	1.07 (1.04 to 1.10)	<0.001	1.05 (1.02 to 1.08)	<0.001	1.06 (1.03 to 1.10)	0.001
4	0.76 ± 0.87	1.25 (1.22 to 1.29)	<0.001	1.12 (1.09 to 1.15)	<0.001	1.09 (1.06 to 1.13)	<0.001	1.11 (1.06 to 1.15)	<0.001
*PM*_*2.5*(*IQR*)_	–	*1.11 (1.10 to 1.13)*	* <0.001*	*1.06 (1.04 to 1.07)*	* <0.001*	*1.04 (1.03 to 1.06)*	* <0.001*	*1.06 (1.04 to 1.07)*	* <0.001*
PM_*10*_									
1	0.68 ± 0.82	Ref		Ref		Ref		Ref	
2	0.71 ± 0.84	1.07 (1.04 to 1.10)	<0.001	1.03 (1.00 to 1.06)	0.037	1.02 (0.99 to 1.05)	0.140	1.01 (0.98 to 1.04)	0.623
3	0.73 ± 0.85	1.10 (1.07 to 1.13)	<0.001	1.05 (1.02 to 1.08)	<0.001	1.04 (1.01 to 1.07)	0.007	1.02 (0.99 to 1.05)	0.228
4	0.72 ± 0.85	1.10 (1.07 to 1.13)	<0.001	1.04 (1.01 to 1.07)	0.005	1.02 (0.99 to 1.05)	0.116	1.00 (0.96 to 1.04)	0.884
*PM*_10(*IQR*)_	–	*1.03 (1.02 to 1.04)*	* <0.001*	*1.01 (1.00 to 1.02)*	*0.005*	*1.01 (0.99 to 1.02)*	*0.142*	*1.00 (0.99 to 1.02)*	*0.758*
PM_coarse_									
1	0.69 ± 0.82	Ref		Ref		Ref		Ref	
2	0.72 ± 0.84	1.05 (1.03 to 1.08)	<0.001	1.03 (1.00 to 1.06)	0.032	1.02 (0.99 to 1.05)	0.089	1.02 (0.99 to 1.05)	0.146
3	0.72 ± 0.85	1.07 (1.04 to 1.10)	<0.001	1.04 (1.01 to 1.07)	0.002	1.03 (1.00 to 1.06)	0.023	1.02 (0.99 to 1.05)	0.101
4	0.72 ± 0.85	1.06 (1.03 to 1.09)	<0.001	1.03 (0.99 to 1.05)	0.055	1.01 (0.98 to 1.04)	0.360	1.00 (0.97 to 1.04)	0.863
*PM*_*coarse*(*IQR*)_	–	*1.01 (1.00 to 1.02)*	*0.011*	*1.00 (0.99 to 1.01)*	*0.288*	*1.00 (0.99 to 1.01)*	*0.806*	*1.01 (0.99 to 1.02)*	*0.560*
NO_2_									
1	0.66 ± 0.80	Ref		Ref		Ref		Ref	
2	0.71 ± 0.84	1.11 (1.08 to 1.14)	<0.001	1.06 (1.03 to 1.09)	<0.001	1.04 (1.01 to 1.07)	0.003	1.05 (1.01 to 1.08)	0.004
3	0.73 ± 0.86	1.17 (1.14 to 1.20)	<0.001	1.08 (1.05 to 1.11)	<0.001	1.06 (1.03 to 1.09)	<0.001	1.07 (1.03 to 1.11)	<0.001
4	0.74 ± 0.87	1.23 (1.19 to 1.26)	<0.001	1.11 (1.07 to 1.14)	<0.001	1.08 (1.05 to 1.11)	<0.001	1.08 (1.04 to 1.13)	<0.001
*NO*_*2*(*IQR*)_	–	*1.11 (1.09 to 1.12)*	* <0.001*	*1.05 (1.04 to 1.07)*	* <0.001*	*1.04 (1.02 to 1.05)*	* <0.001*	*1.04 (1.02 to 1.07)*	* <0.001*

Model 1: Age, sex.

Model 2: Model 1 + ethnicity, education level, employment status, household income.

Model 3: Model 2 + smoking status, alcohol intake frequency, BMI, physical activity.

Model 4: Model 3 + nearest major road, traffic intensity on nearest major road, residential green space (1,000 m) and 24 h noise pollution (Lden).

CI, confidence interval; IQR, interquartile range; NO, nitrogen oxide; PM, particulate matter.

### Air pollution and multimorbidity patterns

In the EFA, 11 multimorbidity patterns emerged with 61.9% of the variance explained. The scree plot and oblique rotated factor loadings are provided in Supplementary Figure 2 and Supplementary Table 7 respectively of the Supplementary Material. Bartlett's Test of Sphericity was significant (*p* < 0.001) and the KMO statistic of 0.54 indicated acceptable sampling adequacy meaning that EFA could be applied to the data. We identified the following patterns (Table 3): cardiovascular; diabetes and hypertension; reproductive organ; common mental health disorders; neurological; gastrointestinal; respiratory; connective tissue, bone, and thyroid; cancer-related multimorbidity; painful conditions; glaucoma-related multimorbidity. In order to be assigned to a multimorbidity pattern, participants had to have at least two of the conditions in the pattern. This meant that participants with multimorbidity could belong to more than one multimorbidity pattern. Any male participant with multimorbidity which comprised prostate conditions and any female participant with multimorbidity which comprised endometriosis were included in the reproductive organ pattern. Any participant with multimorbidity who had cancer was assigned to the cancer-related multimorbidity pattern. Similarly, any participant with multimorbidity who had glaucoma was assigned to the glaucoma-related multimorbidity pattern.

**Table 3:** Multimorbidity patterns identified using exploratory factor analysis (*N* = 156,395).

**Factor**	**Long–term conditions**	**Pattern name**
Factor 1	Atrial fibrillation, CHD, heart failure	Cardiovascular
Factor 2	Diabetes, hypertension, migraine	Diabetes and hypertension
Factor 3	Endometriosis, prostate conditions	Reproductive organ*
Factor 4	Depression, anxiety	Common mental health disorders
Factor 5	Epilepsy, stroke, alcohol/substance dependency	Neurological
Factor 6	Diverticular disease, dyspepsia, IBD, IBS	Gastrointestinal
Factor 7	Asthma, COPD	Respiratory
Factor 8	Connective tissue disorders, osteoporosis, thyroid conditions	Connective tissue, bone, and thyroid
Factor 9	Cancer	Cancer†
Factor 10	Dyspepsia, painful conditions, psoriasis/eczema	Painful conditions
Factor 11	Glaucoma	Glaucoma‡

* Any male participant with multimorbidity which comprised prostate conditions and any female with multimorbidity which comprised endometriosis were included in this pattern.

† Any participant with multimorbidity that had cancer were included in this pattern.

‡ Any participant with multimorbidity that had glaucoma were included in this pattern.

CHD, coronary heart disease; COPD, chronic obstructive pulmonary disorder; IBD, inflammatory bowel disease; IBS, irritable bowel syndrome.

**Figure 2:**
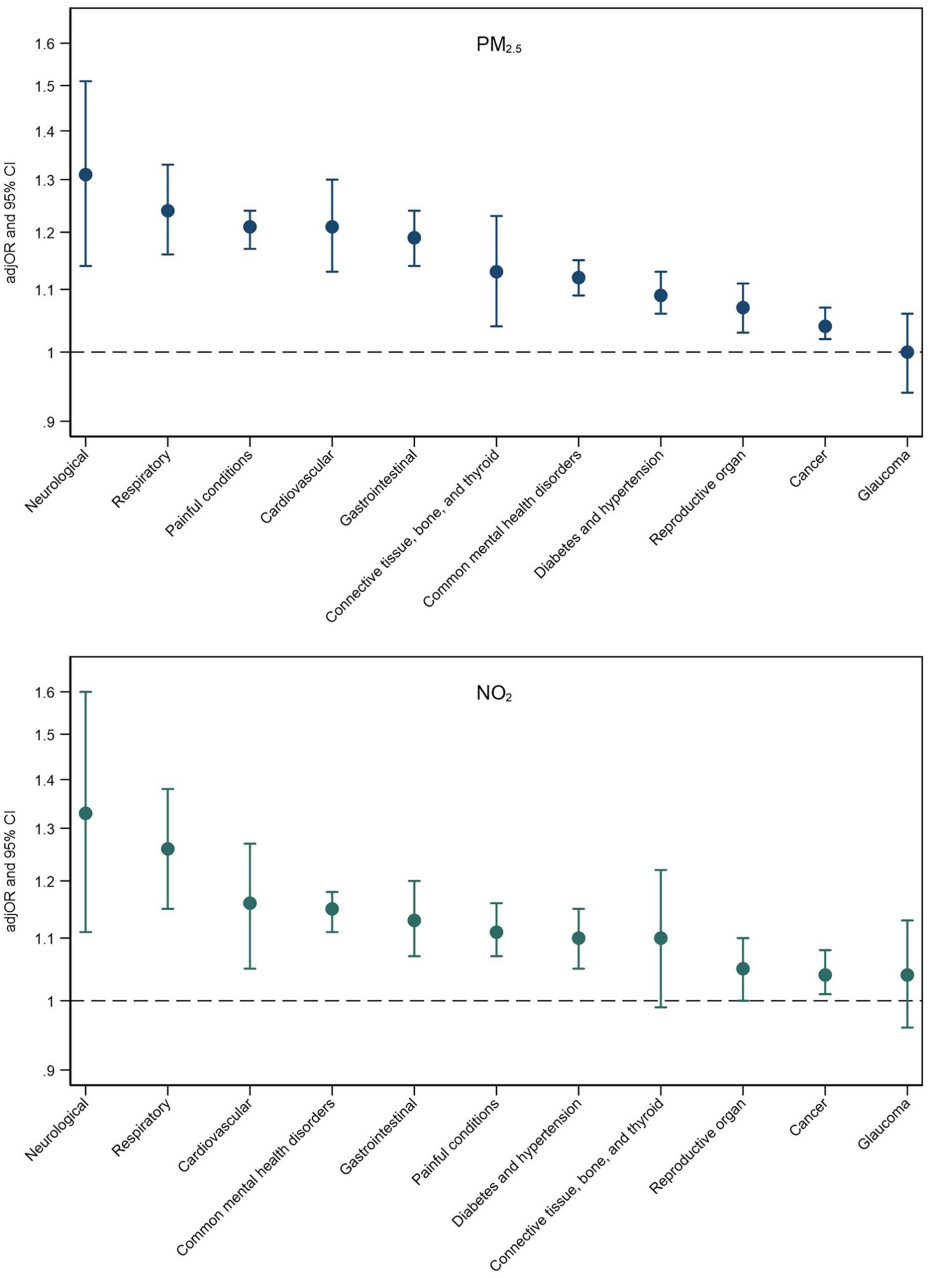
Adjusted odds ratios (adjOR) and their corresponding 95% confidence intervals (CI) from logistic regressions examining associations between PM_2.5_ (top) and NO_2_ (bottom) exposure and the likelihood of belonging to a specific multimorbidity (reference group: those without multimorbidity). All models are adjusted for age, sex, ethnicity, education level, employment status, household income, smoking status, alcohol intake frequency, BMI, physical activity, nearest major road, traffic intensity on nearest major road, residential green space (1,000 m) and 24 h noise pollution (Lden).

Fully adjusted associations between PM_2.5_ and NO_2_ exposure and multimorbidity patterns are presented in Figure 2. Associations for all air pollutants alongside mean multimorbidity severity scores for each pattern are presented in Supplementary Table 8. Higher exposure to PM_2.5_ and NO_2_ was associated with an increased risk of almost all multimorbidity patterns, with strongest associations observed for neurological and respiratory multimorbidity patterns for both air pollutants. Across pollutants, PM_2.5_ exhibited the strongest associations with six multimorbidity patterns (e.g., cardiovascular multimorbidity pattern: adjOR = 1.21, 95% CI = 1.13–1.30; painful conditions: adjOR = 1.21, 95% CI = 1.17–1.24), and NO_2_ exhibited the strongest associations with four patterns (e.g., neurological multimorbidity pattern: adjOR = 1.33, 95% CI = 1.11–1.60; respiratory pattern: adjOR = 1.26, 95% CI = 1.15–1.38).

Exposure to air pollution was strongly associated with neurological and painful multimorbidity patterns. Due to the diversity of conditions included in these patterns, we explored which specific conditions might be the main drivers of these associations. We found that alcohol/substance dependency was the main driver for the neurological pattern (NO_2_ and alcohol/substance dependency: adjOR = 1.73, 95% CI = 1.35–2.20; stroke: adjOR = 1.25, 95% CI = 1.02–1.52; and epilepsy: adjOR = 1.22, 95% CI = 0.98–1.51), and painful conditions were the main driver for the painful multimorbidity pattern (PM_2.5_ and painful conditions: adjOR = 1.21, 95% CI = 1.17–1.25; psoriasis/eczema: adjOR = 1.26, 95% CI = 0.73–2.17; dyspepsia: adjOR = 1.13, 95% CI = 0.84–1.52).

### Sensitivity analyses

Results from the three sensitivity analyses are as follows:

- We looked at associations between air pollution and multimorbidity in participants who completed the baseline assessment in 2010 (*N* = 17,026, 19%) (see Supplementary Table 9). The general pattern of results remained the same as the main analysis.
- Including assessment center as a random variable reduced our effect sizes and 95% CIs slightly for PM_2.5_ and NO_2_, whereas associations with the PM_10_ levels began to show a significant dose-response pattern (Supplementary Table 10).
- Two-pollutant models brought about some changes in the strength and magnitude of some associations. Supplementary Table 11 displays two-way exposure ordinal regression models where each air pollutant was adjusted for in turn to assess potential mutual confounding, which was due to collinear associations between the pollutants (Supplementary Table 3).

## Discussion

In over 360,000 adults from the UK Biobank, we observed a dose-response relationship between exposure to both PM_2.5_ and NO_2_ and multimorbidity status, showing that as air pollution increased, so did the chance of having multimorbid conditions. Moreover, higher exposure to these pollutants was associated with increased severity of multimorbidity amongst those affected. We identified 11 patterns of multimorbidity using exploratory factor analysis. Higher levels of PM_2.5_ and NO_2_ were associated with increased risk of most multimorbidity patterns implying that exposure to air pollution affects multiple body systems. However, these associations differed in strength across the observed patterns indicating that exposure to poor air quality might selectively relate to certain types of multimorbidity patterns more than others, suggesting increased vulnerability for specific population groups.

### Comparison with other studies and potential mechanisms

The results of this study are in line with, and build upon, a previous study which examined associations between PM_2.5_ and the accumulation of LTCs in China (16). Similar to Hu et al., we found that higher PM_2.5_ concentrations were associated with higher levels of multimorbidity, but we also extended these findings to NO_2_, as well as multimorbidity severity. Like Hu et al. (16), we found that higher levels of air pollution were associated with cardiometabolic and respiratory clusters of LTCs, but also found that PM_2.5_ and NO_2_ levels were associated with several other multimorbidity patterns to varying degrees (e.g., neurological and gastrointestinal multimorbidity).

More generally, our findings are in line with large scale studies which have shown that ambient air pollution contributes to global disease burden (31) and specific psychiatric and neurological disorders (8–11). We also found evidence that exposure to air pollution might selectively affect certain multimorbidity patterns, with strongest associations observed for respiratory, cardiovascular, and neurological multimorbidity, as well as the co-occurrence of common mental health disorders. Links between air pollution and respiratory and cardiovascular diseases are well-established (2, 4). Moreover, links between air pollution and depression and anxiety are well reported (9–11) and have been replicated in the current study. We found strong associations between air pollution and neurological multimorbidity which is in line with prior reports linking air pollution and stroke (3), but we found that this association was largely driven by the link between air pollution and alcohol/substance dependency. Previous research has reported that air pollution is associated with increased risk of hospital admission for substance abuse (32). It seems plausible that links between air pollution and alcohol/substance dependency might be a consequence of increased depression and anxiety in those exposed to higher levels of air pollution, given that levels of alcohol/substance dependency are higher in common mental health disorders (33).

In the current study, we observed some less well-established associations. We found that exposure to air pollution increased the likelihood of being in the multimorbidity pattern comprising painful conditions which included conditions such as joint pain, osteoarthritis, and ankylosing spondylitis. Early evidence suggests there are associations between air pollution and pain, with links found between NO_2_ levels and self-rated pain in people with osteoarthritis and spondyloarthritis (34), and PM_2.5_ levels and outpatient visits for osteoarthritis (35). Larger scale mechanistic studies are required to better understand links between air pollution and the development and exacerbation of painful conditions. We also found associations between exposure to air pollution and the gastrointestinal multimorbidity pattern (diverticular disease, dyspepsia, IBD, IBS). Previous research has linked PM_2.5_ and NO_2_ levels with IBS symptoms (36, 37). Links between air pollution and gastrointestinal health require further investigation, but our study is one of the first to provide epidemiological evidence of associations between exposure to air pollution and gastrointestinal diseases.

Air pollution seems to adversely affect several body systems suggesting there may be common pathways through which exposure to pollution might affect health, both in the short- and long-term. There is evidence that exposure to particulate matter and nitrogen dioxide can lead to systemic oxidative stress (38), inflammation (39), and immune activation (40), contributing to pathology across multiple body systems. Disease and organ specific pathways also exist. The inhalational nature of air pollution means that these pollutants deposit in the lungs leading to direct respiratory effects (2). Smaller particulate fractions can translocate into systemic circulation leading to blood coagulation, thrombosis, and cardiovascular events (41). It has also been suggested that particulate pollutants can permeate the blood-brain barrier leading to neuroinflammation (42), which has been implicated in the etiology of mood (43) and other neurological disorders (44). The gastrointestinal tract is highly susceptible to particulate matter and systemic inflammation and oxidative stress associated with exposure to particulate matter may contribute to the damage of the colonic mucosa (45) which might explain links between air pollution and gastrointestinal multimorbidity. Air pollution has been linked to changes in the gut microbiome which might be a potential mechanism through which pollutants induce inflammation in the gastrointestinal tract (46).

We found that higher exposures to PM_2.5_ and NO_2_ were associated with multimorbidity status, severity, and most multimorbidity patterns. However, exposure to PM_10_ and PM_coarse_ was not associated with the presence of multiple LTCs. This is in line with previous research which has shown that particles smaller than 10μm in diameter have the most detrimental effects on human health and have been the ones that have been mainly targeted for air quality mitigation measures (47). A small fraction of fine particles (i.e., PM_2.5_) depositing in the airways can cross the alveolar-capillary barrier, translocating into the circulatory system, to reach peripheral organs in the body (48), potentially exerting deleterious health effects. The association with NO_2_ is less simplistic as it reacts with a range of diverse substrates at the air lung interface and cannot reach the systemic circulation. It is therefore likely that the observed effects reflect the high correlation between NO_2_ and primary combustion particles in ambient air, with the former acting as a proxy of the latter. Coarse particles (i.e., PM_10_ and PM_coarse_) are deposited mainly in the nose and throat and can be eliminated through sneezing and coughing or are swallowed. Therefore, PM_10_ and PM_coarse_ are not fine enough to exert as much damage as other pollutants, which is likely why we observed no association between these air pollutants and multimorbidity outcomes.

### Strengths and limitations

The principal strength of the study was its large sample size of >360,000 and use of multiple evaluated air pollution estimates. We used a high-quality, large UK Biobank cohort with substantial spatial diversity which allowed for considerable variation in levels of air pollution. Further strengths of the study include the use of a validated metric to assess multimorbidity severity and a data-driven approach to identify patterns of multimorbidity, rather than understanding multimorbidity solely in terms of disease count.

Several limitations need consideration. The cross-sectional nature of the study prevents causal inference. Prospective assessments of the association between air pollution and multimorbidity trajectories and patterns are warranted. Air pollution estimates in the current study were from 2010, therefore occurring after the assessment of health status. However, air pollution exposure was relatively stable over this time (2006–10) (49) when baseline assessments were made. We also performed a sensitivity that confirmed the robustness of results. Air pollution estimates were of outdoor concentrations at place of residence and did not include indoor exposures, nor exposure outside of home (e.g., travel, workplace). Further, the study did not have data on the spatial mobility of the participants over the baseline assessment period (2006–2010) meaning we could not account for changes in exposure to air pollution due to change of address. Annual averages of air pollutants meant that variation in these exposures (e.g., seasonal variation) could not be accounted for. Although we included a significant number of confounders in fully adjusted analysis, it is possible there were unmeasured confounders that may bias our results. However, application of the E-methodology confirmed that residual confounding was not likely to significantly affect the estimates. As we included a wide range of sociodemographic, behavioral, and environmental covariates, it is unlikely that there is an unmeasured confounder that would attenuate main associations to non-significant, but it is a possibility that should be borne in mind while interpreting the findings. Nevertheless, the dose-response associations reported in the current study suggest there is a plausible link between air pollution and multimorbidity. The current study sample comprised middle-aged participants resident in England who voluntarily enrolled in the UK Biobank, which may limit generalisability. The UK Biobank cohort is known to differ from the general UK population in terms of sociodemographic (more female, less deprived, fewer ethnic minority participants) and health-related (less smoking, fewer self-reported health conditions) factors (50). Moreover, the exclusion of people with missing data for covariates also poses a major limitation for the interpretation of results due to the introduction of possible selection bias. These data did not appear to be missing at random so were difficult to address using imputation, and resulted in a healthier, wealthier cohort which might affect the generalisability of results.

## Conclusions

In this paper, we have demonstrated a clear association between exposure to air pollution and multimorbidity status, severity, as well as specific multimorbidity patterns. What this suggests is that air pollution might play a role in the accumulation of disease, and that certain organ systems might be more vulnerable to the effects of air pollution than others. These findings provide strong justification and highlight the need for longitudinal studies with repeated measurements of both air pollution and multimorbidity in order to assess prospective associations. Moreover, examining the long-term influence of air pollution on the accumulation of LTCs might help influence health and environmental policy and inform preventative measures to reduce the burden of multimorbidity. Tackling multimorbidity and environmental changes represent the greatest challenges for public health systems worldwide and a deeper more comprehensive understanding of their complex interrelationship is urgently needed (51).

## Data availability statement

Publicly available datasets were analyzed in this study. This data can be requested from the UK Biobank by bona fide researchers for approved projects, including replication, through https://www.ukbiobank.ac.uk/.

## Ethics statement

The studies involving human participants were reviewed and approved by ethical approval for the UK Biobank was granted by the NHS National Research Ethics Service (16/NW/0274). The patients/participants provided their written informed consent to participate in this study.

## Author contributions

AR was involved in the conception and the design of the work, the analysis, the interpretation of the data, and the drafting of the manuscript. JA, AH, MH, IM, and RS were involved in the interpretation of the data and the revision of the paper. MA contributed to the analysis, the interpretation of the data, and the revision of the paper. AD was responsible for data acquisition and contributed to the interpretation of the data and the revision of the paper. IB was involved with the conception and the design of the work, the analysis, the interpretation of the data, and the revision of the paper. All authors contributed to the article and approved the submitted version.

## Funding

AH acknowledges funding from the NIHR Health Protection Research Unit in Environmental Exposures and Health at the University of Leicester, a partnership between UK Health Security Agency, the Health and Safety Executive, and the University of Leicester. IB, RS, and MH are supported by the NIHR Maudsley BRC and IB and RS are supported by the NIHR Collaboration for Leadership in Applied Health Research and Care South London at King's College Hospital NHS Foundation Trust, King's College London. RS is supported by the DATAMIND HDR UK Mental Health Data Hub (MRC grant MR/W014386).

## Conflict of interest

RS declares research support received in the last 36 months from Janssen, GSK, and Takeda. The remaining authors declare that the research was conducted in the absence of any commercial or financial relationships that could be construed as a potential conflict of interest.

## Publisher's note

All claims expressed in this article are solely those of the authors and do not necessarily represent those of their affiliated organizations, or those of the publisher, the editors and the reviewers. Any product that may be evaluated in this article, or claim that may be made by its manufacturer, is not guaranteed or endorsed by the publisher.

## Author disclaimer

The views expressed are those of the author(s) and not necessarily those of the NHS, the NIHR, the Department of Health and Social Care, or the UK Health Security Agency.
