# Supplementary material for: Associations between air pollution and multimorbidity in the UK Biobank: A cross-sectional study
Source: Front Public Health. 2022 Dec 2;10:1035415. doi: 10.3389/fpubh.2022.1035415 (PMC9755180; doi:10.3389/fpubh.2022.1035415)
Supplement: Supplementary file 1 [file Data_Sheet_1.pdf]

Online Supplementary Material

Associations between air pollution and multimorbidity in the UK Biobank: A cross-sectional study

Amy Ronaldson[*1], Jorge Arias de la Torre[1,2,3], Mark Ashworth[4], Anna L Hansell[5,6], Matthew Hotopf[7,8], Ian Mudway[9], Rob Stewart[7,8], Alex Dregan[†7], Ioannis Bakolis[†1,10]

[1]Centre for Implementation Science, Health Service and Population Research Department, Institute of Psychiatry, Psychology and Neuroscience (IoPPN), King's College London, London, United Kingdom

[2]CIBER Epidemiology and Public Health (CIBERESP), Madrid, Spain.

[3]Institute of Biomedicine (IBIOMED), University of Leon, Leon, Spain

[4]School of Life Course and Population Sciences, King's College London, London, United Kingdom

[5]Centre for Environmental Health and Sustainability, University of Leicester, Leicester, United Kingdom

[6]National Institute for Health Research (NIHR) Health Protection Research Unit (HPRU) in Environmental Exposures and Health at the University of Leicester

[7]Department of Psychological Medicine, King's College London, IOPPN and South London and Maudsley NHS Foundation Trust, London, United Kingdom

[8]South London and Maudsley NHS Foundation Trust, London, United Kingdom.

[9]National Institute for Health Research, Health Protection Unit in Environmental Exposures and Health, Imperial College London; and MRC Centre for Environment and Health, School of Public Health, Faculty of Medicine, Imperial College London

[10]Department of Biostatistics and Health Informatics, Institute of Psychiatry, Psychology and Neuroscience, King's College London, London, United Kingdom

*First author

†Joint last author

Contents

1. Supplementary methods ... 3

2. Sample selection (Figure S1) ... 4

3. EFA: Scree plot (Figure S2) ... 5

4. UK Biobank codes for LTCs (Table S1) ... 6

5. Multimorbidity severity weights (Table S2) ... 12

6. Correlation matrix of measures of air pollution (Table S3) ... 13

7. Differences between the analytical sample and excluded participants (Table S4) ... 14

8. Associations between air pollution and multimorbidity status (Table S5) ... 16

9. E-value calculations (Tables S6a and S6b) ... 17

10. Rotated factor loadings (Table S7) ... 20

11. Associations between air pollution and multimorbidity patterns (Table S8) ... 22

12. Associations between air pollution and multimorbidity in participants recruited in 2010 (Table S9) ... 23

13. Associations between air pollution and multimorbidity adjusting for assessment centre location (Table S10) ... 24

14. Associations between air pollutions and multimorbidity – two-way exposure models (Table S11) ... 25

15. Supplementary materials: References ... 26

1. Supplementary methods

Individual-level covariates. Ethnicity [UK Biobank (UKB) field ID 21000] was measured as a categorical variable (White, Asian/Asian British, Black/Black British, Mixed, Other). Education level [UKB field 6138] was coded into three categories: high (college or university degree), intermediate (A/AS levels or equivalent, O levels/GCSEs or equivalent), and low (none of the aforementioned). Employment status [UKB field 6142] was also coded into three categories: employed, retired, unemployed/volunteer/carer. Average total household income before tax [UKB field 738] was coded into five categories (<£18,000, £18,000 to £29,999, £30,000 to £51,999, £52,000 to £100,000, >£100,000). Alcohol intake frequency [UKB field 1558] was measured as a categorical variable (Daily/almost daily, 3-4 times per week, 1-2 times per week, 1-3 times per month, Never/special occasions), as was smoking status [UKB field 20116] (*current smoker, past smoker, never smoked)*. Physical activity [UKB fields 864, 974, 884, 894, 904, 914] was assessed with a modified version of the International Physical Activity Questionnaire that recorded total physical activity (e.g., mild, moderate, vigorous) performed over the previous seven days. Accordingly, participants were classified into four mutually exclusive categories: none, low (<600 metabolic equivalent (MET) minutes/week), moderate (600 to <3000 MET), or vigorous (3000+ MET). Height and weight were collected during the baseline assessment and used to derive BMI [UKB field 21002] using the standard formula (kg/m2) which was then used to derive the following categories: Underweight (<18.5), Normal weight (18.5-24.9), Overweight (25-29.9), Obesity (≥30).

Traffic intensity. Log inverse distance to nearest major road (1/metres) [UKB field 24012] and log traffic intensity on nearest major road (average total number of motor vehicles per 24 hours) [UKB field 24011] were also included as covariates. These were measured in 2010.

Noise exposure. Noise estimates were derived from a simplified version of the Common Noise Assessment Methods in European Union (CNOSSOS-EU) framework[1] which uses land use characteristics including road networks and flows, land cover and meteorology, and the properties of noise propagation from diffraction and refraction, absorption, distance, and angles. These characteristics were used to derive an Lden [UKB field 24024], or a day-evening-night equivalent level, with an A-weighted Leq noise level measured over a 24-h period with a 10 dB penalty added to the levels between 23:00 and 07:00. Noise pollution was modelled in 2009 to place of residence at baseline.

Residential greenspace. Residential green space was also included as a covariate. The percentage of the residential location classed as greenspace was modelled using 2005 data from the Generalised Land Use Database for England (GLUD)[2] for the 2001 Census Output Areas in England. Each residential address was allocated a circular distance buffer of 1000m representing wider area greenspace [UKB field 24500].

2. Sample selection (Figure S1)

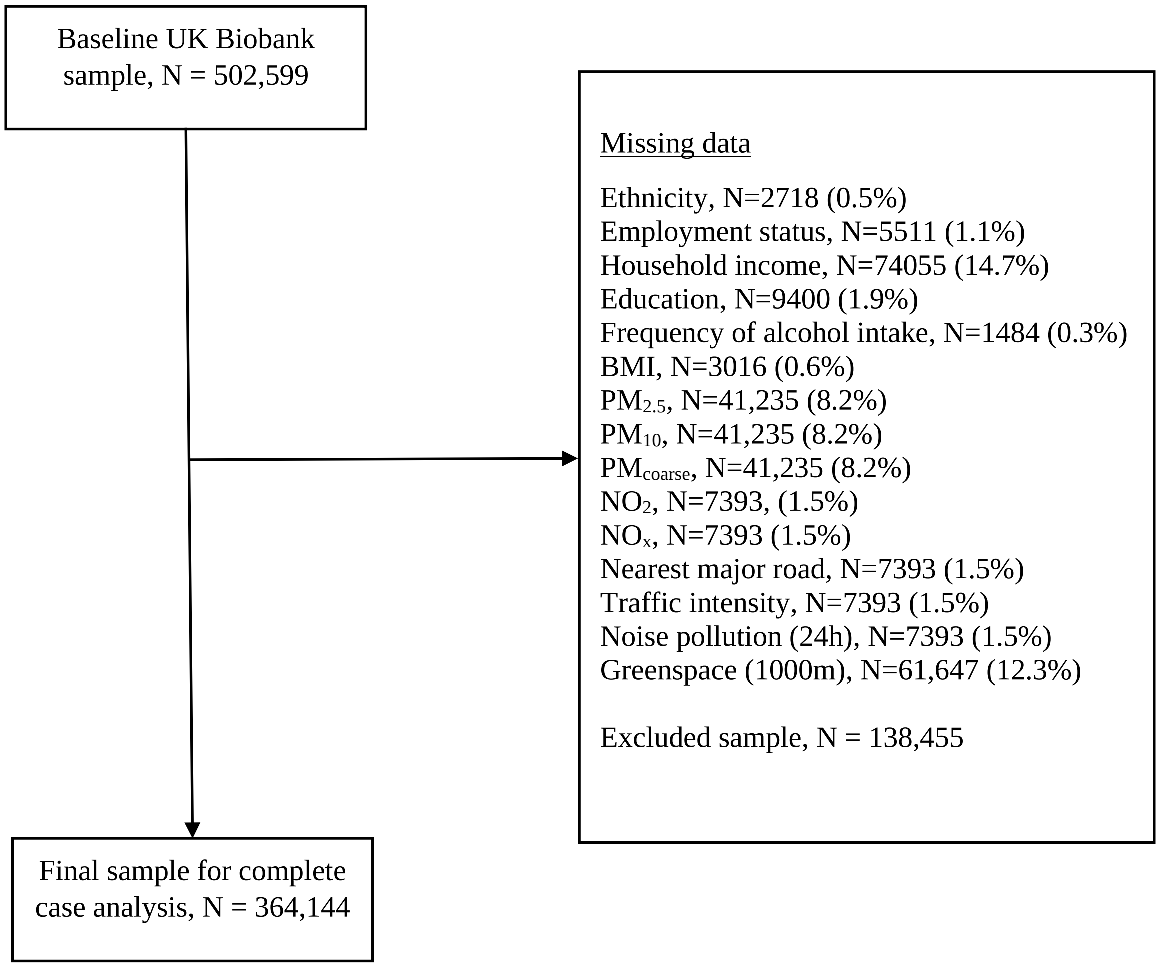

Figure S1. Sample selection flowchart

3. EFA: Scree plot (Figure S2)

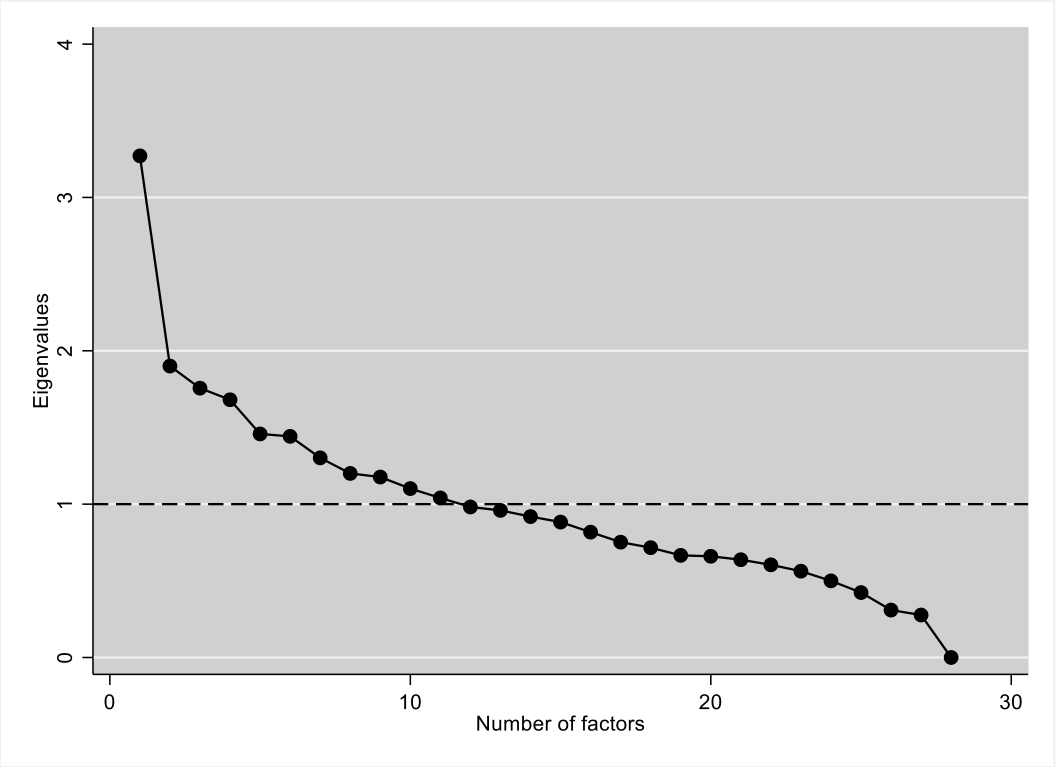

Figure S2. Screeplot indicating the number of factors which emerged (11) following exploratory factor analysis

4. UK Biobank codes for LTCs (Table S1)

Table S1. UK Biobank codes for physical and mental long-term conditions

Condition	*UK Biobank coding (data field 20002)*	*Hospital Episode Statistics (ICD-10 codes)*
Asthma	1111 Asthma	J45 Asthma
Atrial fibrillation	1471 Atrial fibrillation	I48 Atrial fibrillation and flutter<br>I49 Other cardiac arrythmias
Bronchiectasis	1114 Bronchiectasis	J47 Bronchiectasis
Cancer	UK Biobank data field 2453	C17 Malignant neoplasm of small intestine<br>C18 Malignant neoplasm of colon<br>C19 Malignant neoplasm of rectosigmoid junction<br>C20 Malignant neoplasm of rectum<br>C21 Malignant neoplasm of anus and anal canal<br>C16 Malignant neoplasm of stomach<br>C15 Malignant neoplasm of oesophagus<br>C22 Malignant neoplasm of liver and intrahepatic bile ducts<br>C25 Malignant neoplasm of pancreas<br>C50 Malignant neoplasm of breast<br>C34 Malignant neoplasm of bronchus and lung<br>C40 Malignant neoplasm of bone and articular cartilage of limbs<br>C41 Malignant neoplasm of bone and articular cartilage of other and unspecified sites<br>C43 Malignant melanoma of skin<br>C71 Malignant neoplasm of brain<br>C53 Malignant neoplasm of cervix uteri<br>C55 Malignant neoplasm of uterus, part unspecified<br>C56 Malignant neoplasm of ovary<br>C61 Malignant neoplasm of prostate<br>C64 Malignant neoplasm of kidney, except renal pelvis<br>C80 Malignant neoplasm without specification of site<br>C81 Lymphocyte-rich classical Hodgkin lymphoma<br>C82 Follicular lymphoma<br>C83 Non-follicular lymphoma<br>C84 Mature T/NK-cell lymphomas<br>C85 Other specified and unspecified types of non-Hodgkin lymphoma<br>C86 Other specified types of T/NK-cell lymphoma<br>C88 Malignant immunoproliferative diseases and certain other B-cell lymphomas<br>C90 Multiple myeloma and malignant plasma cell neoplasms<br>C91 Lymphoid leukaemia<br>C92 Myeloid leukaemia<br>C93 Monocytic leukaemia<br>C94 Other leukaemias of specified cell type<br>C95 Leukaemia of unspecified cell type<br>C96 Other and unspecified malignant neoplasms of lymphoid, hematopoietic and related tissue<br>C00 Malignant neoplasm of lip<br>C01 Malignant neoplasm of base of tongue

		C02 Malignant neoplasm of other and unspecified parts of tongue<br>C03 Malignant neoplasm of gum<br>C04 Malignant neoplasm of floor of mouth<br>C05 Malignant neoplasm of palate<br>C06 Malignant neoplasm of other and unspecified parts of mouth
Chronic fatigue syndrome (CFS)	1482 Chronic fatigue syndrome	R53.82 Chronic fatigue, unspecified
Chronic kidney disease (CKD)	1192 Renal/kidney failure<br>1193 Renal failure requiring dialysis<br>1194 Renal failure not requiring dialysis<br>1427 Polycystic kidney<br>1519 Kidney nephropathy<br>1520 IGA nephropathy<br>1607 Diabetic nephropathy	N17 Acute kidney failure<br>N18 Chronic kidney disease (CKD)<br>N19 Unspecified kidney failure<br>N00 Acute nephritic syndrome<br>N01 Rapidly progressive nephritic syndrome<br>N03 Chronic nephritic syndrome<br>N04 Nephrotic syndrome<br>N05 Unspecified nephritic syndrome<br>N08 Glomerular disorders in diseases classified elsewhere<br>E08.22 Diabetes mellitus due to underlying condition with diabetic chronic kidney disease<br>E09.22 Drug or chemical induced diabetes mellitus with diabetic chronic kidney disease<br>E10.22 Type 1 diabetes mellitus with diabetic chronic kidney disease<br>E11.22 Type 2 diabetes mellitus with diabetic chronic kidney disease<br>E13.22 Other specified diabetes mellitus with diabetic chronic kidney disease<br>I12 Hypertensive chronic kidney disease<br>I13 Hypertensive heart and chronic kidney disease
Chronic obstructive pulmonary disorder (COPD)	1112 Chronic obstructive airways disease/COPD<br>1113 Emphysema/chronic bronchitis<br>1472 Emphysema	J44 Other chronic obstructive pulmonary disease<br>J43 Emphysema<br>J42 Unspecified chronic bronchitis<br>J41 Simple and mucopurulent chronic bronchitis<br>J40 Bronchitis, not specified as acute or chronic
Chronic sinusitis	1416 Chronic sinusitis	J32 Chronic sinusitis
Connective tissue disorders	1322 Myositis/myopathy<br>1373 Connective tissue disorder<br>1377 Polymyalgia rheumatica<br>1381 Systemic lupus erythematosis/SLE<br>1382 Sjogren's syndrome/sicca syndrome<br>1383 Dermatopolymyositis<br>1384 Scleroderma/systemic sclerosis<br>1456 Malabsorption/coeliac disease<br>1464 Rheumatoid arthritis<br>1477 Psoriatic arthropathy<br>1480 Dermatomyositis<br>1481 Polymyositis	K90 Intestinal malabsorption/coeliac disease<br>M05 Rheumatoid arthritis<br>M06 Other rheumatoid arthritis<br>M07 Psoriatic and enteropathic arthropathies<br>M08 Juvenile arthritis<br>M30 Polyarteritis nodosa and related conditions<br>M31 Other necrotizing vasculopathies<br>M32 Systemic lupus erythematosus<br>M33 Dermatopolymyositis<br>M34 Systemic sclerosis<br>M35 Other systemic involvement of connective tissue<br>M36 Systemic disorders of connective tissue in diseases classified elsewhere<br>M60 Myositis
Coronary heart disease (CHD)	1074 Angina<br>1075 Heart attack/myocardial infarction	I20 Angina pectoris<br>I21 Acute myocardial infarction<br>I22 Subsequent myocardial infarction<br>I23 Certain current complications following acute myocardial infarction<br>I24 Other acute ischaemic heart diseases<br>I25 Chronic ischaemic heart disease

Dementia	1263 Dementia/Alzheimer's/cognitive impairment	F01 Vascular dementia<br>F02 Dementia in other diseases classified elsewhere<br>F03 Unspecified dementia<br>G30 Alzheimer disease<br>G31.83 Dementia with Lewy bodies<br>G31.0 Circumscribed brain atrophy<br>G31.1 Senile degeneration of brain, not elsewhere classified<br>G31.01 Pick's disease<br>G31.09 Other frontotemporal dementia
Diabetes	1220 Diabetes<br>1222 Type 1 diabetes<br>1223 Type 2 diabetes<br>1276 Diabetic eye disease<br>1468 Diabetic neuropathy/ulcers<br>1607 Diabetic nephropathy	E11 Non-insulin-dependent diabetes mellitus<br>E10 Insulin-dependent diabetes mellitus<br>E09 Drug or chemical induced diabetes mellitus<br>E08 Diabetes mellitus due to underlying condition<br>E13 Other specified diabetes mellitus<br>O24.4 Gestational diabetes
Diverticular disease	1458 Diverticular disease/diverticulitis	K57 Diverticular disease of intestine
Dyspepsia/ulcer	1138 Gastro-oesophageal reflux/gastric reflux<br>1139 Oesophagitis/barretts oesophagus<br>1142 Gastric/stomach ulcers<br>1143 Gastritis/gastric erosions<br>1442 Helicobacter pylori<br>1457 Duodenal ulcer<br>1474 Hiatus hernia<br>1510 Dyspepsia/indigestion	K21 Gastro-oesophageal reflux disease<br>K22 Other diseases of oesophagus<br>K25 Gastric ulcer<br>K26 Duodenal ulcer<br>K27 Peptic ulcer, site unspecified<br>K28 Gastrojejunal ulcer<br>K29 Gastritis and duodenitis<br>K30 Dyspepsia
Endometriosis	1402 Endometriosis	N80 Endometriosis
Epilepsy	1264 Epilepsy	G40 Epilepsy
Glaucoma	1277 Glaucoma	H40 Glaucoma<br>H42 Glaucoma in diseases classified elsewhere
Heart failure	1076 Heart failure/pulmonary oedema<br>1079 Cardiomyopathy<br>1588 Hypertrophic cardiomyopathy	I46 Cardiac arrest<br>I50 Heart failure
Hepatitis	1156 Infective/viral hepatitis<br>1578 Hepatitis A<br>1579 Hepatitis B<br>1580 Hepatitis C<br>1581 Hepatitis D<br>1582 Hepatitis E	B15 Acute hepatitis A<br>B16 Acute hepatitis B<br>B17 Other acute viral hepatitis<br>B18 Chronic viral hepatitis<br>B19 Unspecified viral hepatitis
Hypertension	1065 Hypertension<br>1072 Essential hypertension	I10 Essential (primary) hypertension<br>I11 Hypertensive heart disease<br>I12 Hypertensive renal disease<br>I13 Hypertensive heart and renal disease<br>I15 Secondary hypertension
Inflammatory bowel disease (IBD)	1461 Inflammatory bowel disease<br>1462 Crohn's disease<br>1463 Ulcerative colitis	K50 Crohn disease [regional enteritis]<br>K51 Ulcerative colitis<br>K52 Other noninfective gastroenteritis and colitis
Irritable bowel syndrome (IBS)	1154 Irritable bowel syndrome	K58 Irritable bowel syndrome
Liver disease	1141 Oesophageal varicies<br>1157 Non-infective hepatitis<br>1158 Liver failure/cirrhosis<br>1506 Primary biliary cirrhosis	K70 Alcoholic liver disease<br>K71 Toxic liver disease<br>K72 Hepatic failure, not elsewhere classified<br>K73 Chronic hepatitis, not elsewhere classified<br>K74 Fibrosis and cirrhosis of liver

		K75 Other inflammatory liver diseases<br>K76 Other diseases of liver<br>K77 Liver disorders in diseases classified elsewhere
Méniére's disease	1421 Méniére disease	H81.0 Méniére disease
Migraine	1265 Migraine	G43 Migraine
Multiple sclerosis (MS)	1261 Multiple sclerosis	G35 Multiple sclerosis
Osteoporosis	1309 Osteoporosis	M80 Osteoporosis with pathological fracture<br>M81 Osteoporosis without pathological fracture<br>M82 Osteoporosis in diseases classified elsewhere
Painful conditions	1257 Trapped nerve/compressed nerve<br>1294 Back problem<br>1311 Spine arthritis/spondylitis<br>1312 Prolapsed disc/slipped disc<br>1313 Ankylosing spondylitis<br>1436 Headaches (not migraine)<br>1465 Osteoarthritis<br>1466 Gout<br>1476 Sciatica<br>1478 Cervical spondylosis<br>1523 Trigeminal neuralgia<br>1532 Disc problem<br>1533 Disc degeneration<br>1534 Back pain<br>1537 Joint pain<br>1538 Arthritis<br>1540 Plantar fasciitis<br>1541 Carpal tunnel syndrome<br>1542 Fibromyalgia<br>1573 Shingles	M45 Ankylosing spondylitis<br>M46 Other inflammatory spondylopathies<br>M47 Spondylosis<br>M48 Other spondylopathies<br>M49 Spondylopathies in diseases classified elsewhere<br>M50 Cervical disc disorders<br>M51 Other intervertebral disc disorders<br>M53 Other dorsopathies, not elsewhere classified<br>M54 Dorsalgia<br>R51 Headache<br>G44 Other headache syndromes<br>M10 Gout<br>M11 Other crystal arthropathies<br>M12 Other specific arthropathies<br>M13 Other arthritis<br>M14 Arthropathies in other diseases classified elsewhere<br>M72.2 Plantar fasciitis<br>G50.0 Trigeminal neuralgia<br>G50.1 Atypical facial pain<br>G56.0 Carpal tunnel syndrome<br>M79.7 Fibromyalgia<br>B02 Herpes zoster (shingles)
Parkinson's disease	1262 Parkinson's disease	G20 Parkinson's disease<br>G21 Secondary parkinsonism
Peripheral vascular disease (PVD)	1067 Peripheral vascular disease<br>1087 Leg claudication/intermittent claudication	I700 Atherosclerosis of aorta<br>I702 Atherosclerosis of arteries of extremities<br>I708 Atherosclerosis of other arteries<br>I709 Generalized and unspecified atherosclerosis<br>I731 Thromboangiitis obliterans [Buerger]<br>I738 Other specified peripheral vascular diseases<br>I739 Peripheral vascular disease, unspecified
Pernicious anaemia	1331 Pernicious anaemia	D51.0 Vitamin B12 deficiency anaemia due to intrinsic factor deficiency
Polycystic ovarian syndrome (PCOS)	1350 Polycystic ovarian syndrome	E28.2 Polycystic ovarian syndrome
Prostate conditions (not cancer)	1207 Prostate problem (not cancer)<br>1396 Enlarged prostate<br>1516 Benign prostatic hypertrophy	N40 Hyperplasia of prostate<br>N41 Inflammatory diseases of prostate<br>N42 Other disorders of prostate
Psoriasis/eczema	1452 Eczema/dermatitis<br>1453 Psoriasis	L20 Atopic dermatitis<br>L21 Seborrhoeic dermatitis<br>L22 Diaper [napkin] dermatitis<br>L23 Allergic contact dermatitis<br>L24 Irritant contact dermatitis<br>L25 Unspecified contact dermatitis<br>L26 Exfoliative dermatitis

		L27 Dermatitis due to substances taken internally<br>L28 Lichen simplex chronicus and prurigo<br>L29 Pruritis<br>L30 Other and unspecified dermatitis<br>L40 Psoriasis<br>L41 Parapsoriasis
Stroke/transient ischaemic attack (TIA)	1081 Stroke<br>1082 Transient ischaemic attack<br>1086 Subarachnoid haemorrhage<br>1491 Brain haemorrhage<br>1583 Ischaemic stroke	I60 Subarachnoid haemorrhage<br>I61 Intracerebral haemorrhage<br>I62 Other nontraumatic intracranial haemorrhage<br>I63 Cerebral infarction<br>I65 Occlusion and stenosis of precerebral arteries, not resulting in cerebral infarction<br>I66 Occlusion and stenosis of cerebral arteries, not resulting in cerebral infarction<br>I67 Other cerebrovascular diseases<br>I68 Cerebrovascular disorders in diseases classified elsewhere<br>I69 Sequelae of cerebrovascular disease
Thyroid conditions	1224 Thyroid problem (not cancer)<br>1225 Hyperthyroidism/thyrotoxicosis<br>1226 Hypothyroidism/myxoedema<br>1428 Thyroiditis<br>1522 Grave's disease<br>1610 Thyroid goitre	E00 Congenital iodine-deficiency syndrome<br>E01 Iodine-deficiency-related thyroid disorders and allied conditions<br>E02 Subclinical iodine-deficiency hypothyroidism<br>E03 Other hypothyroidism<br>E04 Other nontoxic goitre<br>E05 Thyrotoxicosis [hyperthyroidism]<br>E06 Thyroiditis<br>E07 Other disorders of thyroid
Depression	1286 Depression<br>1531 Post-natal depression<br>*+ Patient Health Questionnaire (PHQ)-4 depression items*	F32 Depressive episode<br>F33 Recurrent depressive disorder<br>F34 Persistent mood disorders<br>F38 Other mood disorders<br>F39 Unspecified mood disorder
Anxiety	1287 Anxiety/panic attacks<br>1615 Obsessive compulsive disorder (OCD)<br>1469 Post-traumatic stress disorder (PTSD)<br>1614 Stress<br>*+ PHQ-4 anxiety items*	F41 Other anxiety disorders<br>F42 Obsessive-compulsive disorder<br>F431 Post-traumatic stress disorder
Alcohol/substance dependency	1408 Alcohol dependency<br>1604 Alcoholic liver disease/alcoholic cirrhosis<br>1409 Opioid dependency<br>1410 Other substance abuse/dependency	F10 Mental and behavioural disorders due to use of alcohol<br>F11 Mental and behavioural disorders due to use of opioids<br>F12 Mental and behavioural disorders due to use of cannabinoids<br>F13 Mental and behavioural disorders due to use of sedatives or hypnotics<br>F14 Mental and behavioural disorders due to use of cocaine<br>F15 Mental and behavioural disorders due to use of other stimulants, including caffeine<br>F16 Mental and behavioural disorders due to use of hallucinogens<br>F19 Mental and behavioural disorders due to multiple drug use and use of other psychoactive substances
Severe mental illness (SMI)	1289 Schizophrenia<br>1291 Mania/bipolar disorder/manic depression	F20 Schizophrenia<br>F21 Schizotypal disorder<br>F22 Persistent delusional disorder

		F23 Acute and transient psychotic disorders<br>F24 Induced delusional disorder<br>F25 Schizoaffective disorder<br>F28 Other nonorganic psychotic disorders<br>F29 Unspecified nonorganic psychosis<br>F30 Manic episode<br>F31 Bipolar affective disorder
Anorexia or bulimia	1470 Anorexia/bulimia/other eating disorder	F50 Eating disorders

5. Multimorbidity severity weights (Table S2)

Table S2. Established severity weights from the Cambridge Multimorbidity Score based on health service use and mortality data for each condition included in the multimorbidity measure. Health status based on a classification developed by the Reinsurance Group of America is provided for each condition.

Condition	*Severity weight*	*Health classification*
Asthma	0.18	Healthy
Atrial fibrillation	1.30	Unhealthy
Bronchiectasis	0.66	Healthy
Cancer	1.50	Unhealthy
Chronic fatigue syndrome (CFS)*	-0.10	Healthy
Chronic kidney disease (CKD)	0.51	Unhealthy
Chronic obstructive pulmonary disorder (COPD)	1.41	Unhealthy
Chronic sinusitis	0.13	Healthy
Connective tissue disorders	0.40	Unhealthy
Coronary heart disease (CHD)	0.46	Unhealthy
Dementia	2.46	Unhealthy
Diabetes	0.71	Unhealthy
Diverticular disease	-0.02	Healthy
Dyspepsia/ulcer	0.20	Healthy
Endometriosis*	0.05	Healthy
Epilepsy	0.85	Unhealthy
Glaucoma*	0.44	Healthy
Heart failure	1.12	Unhealthy
Hepatitis*	0.67	Unhealthy
Hypertension	0.09	Healthy
Inflammatory bowel disease (IBD)	0.44	Healthy
Irritable bowel syndrome (IBS)	0.18	Healthy
Liver disease	0.72	Unhealthy
Méniére’s disease*	0.36	Healthy
Migraine	0.07	Healthy
Multiple sclerosis (MS)	0.69	Unhealthy
Osteoporosis*	0.76	Healthy
Painful conditions	0.87	
Parkinson’s disease	1.29	Unhealthy
Peripheral vascular disease (PVD)	0.53	Unhealthy
Pernicious anaemia*	0.97	Unhealthy
Polycystic ovarian syndrome (PCOS)*	-1.02	Healthy
Prostate conditions (not cancer)	0.01	Healthy
Psoriasis/eczema	0.25	Healthy
Stroke/transient ischaemic attack (TIA)	0.77	Unhealthy
Thyroid conditions	0.08	Healthy
Depression	0.47	Unhealthy
Anxiety	0.47	Unhealthy
Alcohol problems and other psychoactive substance abuse	0.47**	Unhealthy
Severe mental illness	0.58	Unhealthy
Anorexia or bulimia	0.34	Unhealthy

*Imputed severity weights

**Average of severity weight for alcohol problems (0.55) and substance abuse (0.38)

6. Correlation matrix of measures of air pollution (Table S3)

Table S3. Correlation matrix of measures of air pollution in UK Biobank (Pearson correlations)

Environmental exposure	M±SD	IQR	Pearson correlation coefficients*			
			$PM_{2.5}$	PM_{10}	PM_{coarse}	NO_2
$PM_{2.5}$ (μg/m³)	9.98±1.05	1.27	1			
PM_{10} (μg/m³)	16.21±1.88	1.75	0.539	1		
PM_{coarse} (μg/m³)	6.41±0.89	0.78	0.221	0.812	1	
NO_2 (μg/m³)	26.60±7.61	9.90	0.866	0.510	0.200	1

IQR=interquartile range; M=mean; OR= odds ratio; PM=particulate matter; S=standard deviation
*All coefficients have a p-value <0.0001

7. Differences between the analytical sample and excluded participants (Table S4)

Table S4. Differences between the analytical sample (N=364,411) and excluded participants (N=138,455) from the UK Biobank

	Analytical sample (N=364,144, 72.5%)	Excluded participants (N=138,455, 27.5%)	
	M±SD or *N(%)*	*M±SD* or *N(%)*	*P-value*
Age	56.24±8.08	57.28±8.08	<0.001
Female	191,563 (52.6)	81,881 (59.1)	<0.001
Ethnicity			<0.001
White	346,026 (95.0)	126,773 (93.4)	
Asian/Asian British	6362 (1.8)	3509 (2.6)	
Black/Black British	5607 (1.5)	2452 (1.8)	
Mixed	2147 (0.6)	819 (0.6)	
Other	4002 (1.1)	2184 (1.6)	
Education level			<0.001
High	110,330 (30.3)	32,267 (25.0)	
Intermediate	81,231 (22.3)	23,957 (18.6)	
Low	172,583 (47.4)	72,831 (56.4)	
Employment status			<0.001
Employed	221,091 (60.7)	51,711 (38.9)	
Retired	115,445 (31.7)	66,186 (49.8)	
Unemployed/volunteer/carer	27,608 (7.6)	15,047 (11.3)	
Household income			<0.001
<£18,000	79,952 (22.0)	15,513 (24.1)	
£18,000 to £29,999	92,318 (23.4)	15,873 (24.6)	
£30,000 to £51,999	95,415 (26.2)	16,651 (25.9)	
£52,000 to £100,000	75,670 (20.8)	12,985 (20.2)	
>£100,000	20,789 (5.7)	3378 (5.2)	
Alcohol intake frequency			<0.001
Daily/almost daily	77,939 (21.4)	23,848 (17.4)	

3-4 times per week	86,857 (23.9)	28,600 (20.9)	
1-2 times per week	92,935 (25.5)	36,381 (26.6)	
1-3 times per month	40,453 (11.1)	15,418 (11.3)	
Never/special occasions	65,960 (18.1)	32,724 (23.9)	
Current or past smoker	165,916 (45.6)	60,805 (43.9)	<0.001
Physical activity			<0.001
None	5952 (1.6)	5803 (4.2)	
Low	67,868 (18.6)	31,346 (22.6)	
Moderate	148,427 (40.8)	55,880 (40.4)	
Vigorous	141,897 (39.0)	45,426 (32.8)	
BMI (kg/m^2)			<0.001
Underweight (<18.5)	1835 (0.5)	792 (0.6)	
Normal weight (18.5-24.9)	119,812 (32.9)	42,658 (31.5)	
Overweight (25-29.9)	155,518 (42.7)	56,662 (41.8)	
Obesity (≥30)	86,979 (23.9)	35,327 (26.1)	
Nearest major road (1/metres)	0.006±0.016	0.006±0.017	0.390
Traffic intensity on nearest major road (vehicles/day)	23,194.3±20,908.2	24,933.9±22,073.4	<0.001
Noise pollution 24h (dB)	56.04±4.27	56.14±4.31	<0.001
Residential greenspace 1000m (%)	45.28±21.64	43.60±21.51	<0.001
$PM_{2.5}$ ($\mu g/m^3$)	9.98±1.05	10.04±1.09	<0.001
PM_{10} ($\mu g/m^3$)	16.20±1.88	16.36±1.97	<0.001
PM_{coarse} ($\mu g/m^3$)	6.41±0.89	6.49±0.95	<0.001
NO_2 ($\mu g/m^3$)	26.60±7.61	27.04±7.48	<0.001
Number of conditions			
0 or 1 condition (no MM)	207,749 (57.1)	70,881 (51.2)	
2 conditions	75,689 (20.8)	29,895 (21.6)	
3 conditions	42,611 (11.7)	18,459 (13.3)	
4 or more conditions	38,095 (10.5)	19,220 (13.9)	
Multimorbidity severity score	0.71±0.84	0.82±0.91	<0.001

8. Associations between air pollution and multimorbidity status (Table S5)

Table S5. Associations between IQR increments in air pollution (*italics*), air pollution quartiles[a] and multimorbidity in single exposure ordinal logistic regression models (N=364,144)

	Model 1		Model 2		Model 3		Model 4	
	OR (95% CI)	*p value*	*OR (95% CI)*	*p value*	*OR (95% CI)*	*p value*	*OR (95% CI)*	*p value*
$PM_{2.5}$								
1	Ref		Ref		Ref		Ref	
2	1.14 (1.12 to 1.17)	<0.001	1.07 (1.06 to 1.10)	<0.001	1.05 (1.03 to 1.07)	<0.001	1.08 (1.06 to 1.10)	<0.001
3	1.25 (1.23 to 1.27)	<0.001	1.12 (1.10 to 1.14)	<0.001	1.08 (1.06 to 1.10)	<0.001	1.13 (1.10 to 1.15)	<0.001
4	1.39 (1.37 to 1.42)	<0.001	1.19 (1.17 to 1.21)	<0.001	1.14 (1.12 to 1.16)	<0.001	1.21 (1.18 to 1.24)	<0.001
$PM_{2.5\ (IQR)}$	*1.17 (1.16 to 1.18)*	*<0.001*	*1.08 (1.07 to 1.09)*	*<0.001*	*1.06 (1.05 to 1.07)*	*<0.001*	*1.10 (1.08 to 1.11)*	*<0.001*
PM_{10}								
1	Ref		Ref		Ref		Ref	
2	1.10 (1.08 to 1.12)	<0.001	1.04 (1.02 to 1.06)	<0.001	1.03 (1.01 to 1.04)	0.008	1.02 (1.00 to 1.04)	0.105
3	1.16 (1.13 to 1.18)	<0.001	1.07 (1.05 to 1.09)	<0.001	1.05 (1.03 to 1.07)	<0.001	1.03 (1.01 to 1.06)	0.002
4	1.16 (1.14 to 1.18)	<0.001	1.07 (1.05 to 1.09)	<0.001	1.04 (1.02 to 1.06)	<0.001	1.02 (1.00 to 1.05)	0.092
$PM_{10\ (IQR)}$	*1.06 (1.05 to 1.06)*	*<0.001*	*1.03 (1.02 to 1.03)*	*<0.001*	*1.02 (1.01 to 1.02)*	*<0.001*	*1.02 (1.01 to 1.03)*	*0.002*
PM_{coarse}								
1	Ref		Ref		Ref		Ref	
2	1.09 (1.08 to 1.11)	<0.001	1.06 (1.04 to 1.07)	<0.001	1.04 (1.03 to 1.06)	<0.001	1.04 (1.02 to 1.06)	<0.001
3	1.11 (1.09 to 1.13)	<0.001	1.06 (1.04 to 1.08)	<0.001	1.04 (1.02 to 1.06)	<0.001	1.04 (1.02 to 1.06)	<0.001
4	1.12 (1.10 to 1.14)	<0.001	1.06 (1.04 to 1.08)	<0.001	1.04 (1.02 to 1.06)	<0.001	1.03 (1.00 to 1.05)	0.022
$PM_{coarse\ (IQR)}$	*1.02 (1.02 to 1.03)*	*<0.001*	*1.01 (1.01 to 1.02)*	*<0.001*	*1.01 (1.00 to 1.01)*	*0.014*	*1.01 (0.99 to 1.02)*	*0.300*
NO_2								
1	Ref		Ref		Ref		Ref	
2	1.18 (1.16 to 1.20)	<0.001	1.09 (1.07 to 1.11)	<0.001	1.06 (1.04 to 1.08)	<0.001	1.09 (1.07 to 1.11)	<0.001
3	1.27 (1.25 to 1.29)	<0.001	1.13 (1.11 to 1.15)	<0.001	1.08 (1.06 to 1.10)	<0.001	1.15 (1.12 to 1.17)	<0.001
4	1.34 (1.31 to 1.36)	<0.001	1.15 (1.13 to 1.17)	<0.001	1.10 (1.08 to 1.12)	<0.001	1.20 (1.16 to 1.23)	<0.001
$NO_{2\ (IQR)}$	*1.15 (1.14 to 1.16)*	*<0.001*	*1.07 (1.06 to 1.08)*	*<0.001*	*1.05 (1.04 to 1.06)*	*<0.001*	*1.10 (1.08 to 1.11)*	*<0.001*

Model 1: Age, sex
Model 2: Model 1 + ethnicity, education level, employment status, household income
Model 3: Model 2 + smoking status, alcohol intake frequency, BMI, physical activity
Model 4: Model 3 + nearest major road, traffic intensity on nearest major road, residential green space (1000m) and 24h noise pollution (Lden)
a.The quartile cut-offs for NO_2 were: Q1 <21.32 μg/m^3, Q2 <26.09 μg/m^3, Q3 <31.22 μg/m^3 and Q4 ≥31.22 μg/m^3; for $PM_{2.5}$ they were: Q1 <9.29 μg/m^3, Q2 <9.93 μg/m^3, Q3 <10.56 μg/m^3 and Q4 ≥10.56 μg/m^3; for PM_{coarse} they were: Q1 <5.85 μg/m^3, Q2 <6.11 μg/m^3, Q3 <6.63 μg/m^3 and Q4 ≥6.63 μg/m^3; and for PM_{10} they were: Q1 <15.24 μg/m^3, Q2 <16.03 μg/m^3, Q3 <16.99 μg/m^3 and Q4 ≥16.99 μg/m^3.
CI=confidence interval; IQR=interquartile range; OR=odds ratio; PM=particulate matter

9. E-value calculations (Tables S6a and S6b)

Table S6a. Fully-adjusted (model 5) cross-sectional associations between PM2.5 and multimorbidity status with E-values

	OR (95% CI)	*p value*	*E-value*	*Lower CI limit**
$PM_{2.5}$				
Q1	Ref			
Q2	1.08 (1.06 to 1.10)	<0.001	1.37	1.31
Q3	1.13 (1.10 to 1.15)	<0.001	1.51	1.43
Q4	1.21 (1.18 to 1.24)	<0.001	1.71	1.64
$PM_{2.5\ (IQR)}$	1.10 (1.08 to 1.11)	<0.001	1.43	1.37
Age	1.04 (1.03 to 1.04)	<0.001		
Sex				
Female	Ref			
Male	0.92 (0.90 to 0.93)	<0.001		
Ethnicity				
White	Ref			
Asian/Asian British	1.52 (1.45 to 1.60)	<0.001		
Black/Black British	1.00 (0.95 to 1.06)	0.795		
Mixed	1.03 (0.94 to 1.12)	0.565		
Other	1.03 (0.96 to 1.09)	0.403		
Education level				
Low	Ref			
Intermediate	1.07 (1.05 to 1.09)	<0.001		
High	1.05 (1.03 to 1.06)	<0.001		
Employment status				
Unemployed/volunteer/carer	Ref			
Employed	0.43 (0.42 to 0.44)	<0.001		
Retired	0.52 (0.50 to 0.53)	<0.001		
Household income				
<£18,000	Ref			
£18,000 to £29,999	0.72 (0.70 to 0.73)	<0.001		
£30,000 to £51,999	0.61 (0.59 to 0.62)	<0.001		
£52,000 to £100,000	0.50 (0.49 to 0.51)	<0.001		
>£100,000	0.41 (0.39 to 0.42)	<0.001		
Alcohol intake frequency				
Daily/almost daily	Ref			
3-4 times per week	0.94 (0.92 to 0.96)	<0.001		
1-2 times per week	1.03 (1.01 to 1.05)	0.001		
1-3 times per month	1.19 (1.16 to 1.22)	<0.001		
Special occasions/Never	1.58 (1.54 to 1.62)	<0.001		
Smoking status				
Never smoked	Ref			
Past smoker	1.28 (1.26 to 1.29)	<0.001		
Current smoker	1.32 (1.29 to 1.35)	<0.001		
Physical activity				
None	Ref			
Low	0.51 (0.49 to 0.54)	<0.001		
Moderate	0.41 (0.39 to 0.43)	<0.001		

Vigorous	0.35 (0.33 to 0.36)	<0.001
BMI (kg/m^2)		
Normal weight (18.5-24.9)	Ref	
Underweight (<18.5)	1.17 (1.06 to 1.28)	0.001
Overweight (25-29.9)	1.35 (1.33 to 1.38)	<0.001
Obesity (≥30)	2.33 (2.29 to 2.37)	<0.001
Nearest major road (1/metres)	1.19 (0.74 to 1.91)	0.477
Traffic intensity on nearest major road (vehicles/day)	1.00 (0.99 to 1.00)	0.583
Noise pollution 24h (Lden)	0.99 (0.99 to 1.00)	<0.001
Residential greenspace 1000m (%)	1.00 (1.00 to 1.01)	<0.001

*E-values do not include p-values but the lower CI is above 1 (i.e., robust)
BMI=body mass index; dB=decibel; CI=confidence interval; IQR=interquartile range; OR=odds ratio; PM=particulate matter

Table S6b. Fully-adjusted cross-sectional associations between NO2 and multimorbidity status with E-values

	OR (95% CI)	*p value*	*E-value*	*Lower CI limit**
NO_2				
Q1	Ref			
Q2	1.09 (1.07 to 1.11)	<0.001	1.40	1.34
Q3	1.15 (1.12 to 1.17)	<0.001	1.57	1.49
Q4	1.20 (1.16 to 1.23)	<0.001	1.69	1.59
$NO_{2\ (IQR)}$	1.07 (1.06 to 1.08)	<0.001	1.43	1.37
Age	1.04 (1.03 to 1.04)	<0.001		
Sex				
Female	Ref			
Male	0.92 (0.90 to 0.93)	<0.001		
Ethnicity				
White	Ref			
Asian/Asian British	1.49 (1.42 to 1.57)	<0.001		
Black/Black British	0.99 (0.94 to 1.04)	0.841		
Mixed	1.02 (0.93 to 1.11)	0.673		
Other	1.02 (0.95 to 1.08)	0.571		
Education level				
Low	Red			
Intermediate	1.07 (1.05 to 1.09)	<0.001		
High	1.05 (1.03 to 1.06)	<0.001		
Employment status				
Unemployed/volunteer/carer	Ref			
Employed	0.44 (0.42 to 0.45)	<0.001		
Retired	0.52 (0.50 to 0.53)	<0.001		
Household income				
<£18,000	Ref			
£18,000 to £29,999	0.72 (0.70 to 0.73)	<0.001		
£30,000 to £51,999	0.60 (0.59 to 0.62)	<0.001		
£52,000 to £100,000	0.50 (0.48 to 0.51)	<0.001		
>£100,000	0.40 (0.39 to 0.42)	<0.001		
Alcohol intake frequency				
Daily/almost daily	Ref			

3-4 times per week	0.94 (0.92 to 0.96)	<0.001
1-2 times per week	1.03 (1.01 to 1.05)	0.001
1-3 times per month	1.19 (1.16 to 1.22)	<0.001
Special occasions/never	1.58 (1.55 to 1.62)	<0.001
Smoking status		
Never smoked	Ref	
Past smoker	1.28 (1.26 to 1.29)	<0.001
Current smoker	1.32 (1.29 to 1.35)	<0.001
Physical activity		
None	Ref	
Low	0.51 (0.49 to 0.54)	<0.001
Moderate	0.41 (0.39 to 0.43)	<0.001
Vigorous	0.35 (0.33 to 0.36)	<0.001
BMI (kg/m^2)		
Normal weight (18.5-24.9)	Ref	
Underweight (<18.5)	1.17 (1.06 to 1.28)	0.001
Overweight (25-29.9)	1.35 (1.33 to 1.38)	<0.001
Obesity (≥30)	2.33 (2.29 to 2.37)	<0.001
Nearest major road (1/metres)	1.30 (0.81 to 2.10)	0.278
Traffic intensity on nearest major road (vehicles/day)	1.00 (1.00 to 1.00)	0.109
Noise pollution 24h (Lden)	1.00 (0.99 to 1.00)	<0.001
Residential greenspace 1000m (%)	1.00 (1.00 to 1.00)	<0.001

*E-values do not include p-values but the lower CI is above 1 (i.e., robust)

BMI=body mass index; CI=confidence interval; IQR=interquartile range; OR=odds ratio; PM=particulate matter

10. Rotated factor loadings (Table S7)

Table S7. Rotated factor loadings for the 11 factors that emerged from exploratory factor analysis on physical and mental long-term conditions

	Factor1	Factor2	Factor3	Factor 4	Factor5	Factor6	Factor7	Factor8	Factor9	Factor10	Factor11	Uniqueness
Asthma	-0.0943	-0.0521	0.123	0.1985	-0.0527	-0.1059	**0.7268**	-0.0946	0.0426	-0.2734	-0.0088	0.3271
Atrial fibrillation	**0.8838**	-0.2043	-0.0308	0.1001	-0.0076	-0.0633	-0.0584	-0.0661	-0.041	-0.0373	-0.0007	0.317
Cancer	-0.0998	-0.0911	-0.0369	0.2126	-0.1080	-0.0678	-0.0536	-0.0816	**-0.9605**	-0.0059	0.0742	0.1736
CKD	0.3105	0.3503	0.0544	0.0611	0.0608	0.1201	-0.0702	0.1727	-0.1215	-0.1176	0.2744	0.4749
COPD	0.0970	-0.0352	-0.0083	-0.0008	0.0122	-0.0249	**0.7760**	0.1073	0.0504	0.1371	0.0235	0.3575
CTD	-0.0518	0.0182	-0.0958	0.0293	-0.0772	0.0095	-0.0328	**0.8126**	0.2662	-0.1539	0.1257	0.299
CHD	**0.6595**	0.1718	-0.051	0.0343	-0.0217	0.0213	0.0669	-0.0478	0.1857	0.0678	-0.0957	0.415
Diabetes	-0.0480	**0.8111**	0.0461	0.0370	-0.0649	-0.0598	-0.0101	-0.1203	0.1188	-0.0369	-0.0268	0.3938
Diverticular disease	-0.0258	0.0505	0.0001	0.1309	-0.0769	**0.6499**	0.0209	-0.0293	0.0730	0.1832	-0.3111	0.4977
Dyspepsia	-0.0611	-0.0326	-0.0626	0.0595	-0.0547	**0.4341**	0.1253	0.0354	0.0967	**0.4322**	0.0708	0.6158
Epilepsy	-0.1202	-0.1406	0.0122	0.0465	**0.8607**	-0.0814	-0.0419	0.0099	0.0550	-0.0914	-0.2233	0.3063
Endometriosis	0.0006	-0.0312	**0.9252**	0.0517	-0.0546	0.0687	-0.0199	-0.1018	-0.0123	-0.0091	0.0242	0.1174
Glaucoma	0.0203	0.0081	-0.0624	0.1372	0.1129	0.0633	0.0060	-0.0733	0.0575	-0.132	**-0.8242**	0.3587
Hypertension	-0.1227	**0.8212**	0.0045	0.2494	-0.0062	-0.1316	-0.1064	-0.1097	0.0436	0.1082	0.1053	0.2998
Heart failure	**0.9283**	-0.0612	0.0422	-0.0118	-0.0067	-0.0131	0.0584	-0.0428	0.0525	-0.0401	0.0181	0.2058
IBD	0.0365	-0.0150	0.0188	0.0363	0.0016	**0.6492**	-0.0908	0.0804	-0.1657	-0.1745	0.1124	0.4355
IBS	-0.0488	-0.2528	0.1046	0.0098	-0.0534	**0.6114**	-0.1358	-0.1103	0.2086	0.0290	0.0003	0.4911
Migraine	-0.0374	**-0.4550**	0.0997	0.1492	0.0921	-0.0556	-0.3466	-0.0536	0.0922	0.0437	0.1602	0.5108
Osteoporosis	-0.0991	-0.2011	0.0308	0.1010	0.1473	0.0148	0.1254	**0.6499**	-0.1861	0.1486	0.0008	0.4204
Painful conditions	-0.0978	-0.0790	-0.0172	0.2173	0.0133	-0.0192	-0.0078	0.0217	0.1886	**0.5848**	0.2304	0.5314
Prostate conditions	0.0152	-0.0730	**-0.9606**	0.0545	-0.0733	0.0096	-0.0930	-0.0759	-0.0549	-0.0103	-0.0447	0.0816
Psoriasis/eczema	-0.0811	-0.0840	-0.0628	0.1836	-0.0214	-0.0209	0.0985	0.0490	0.2726	**-0.6562**	0.0533	0.3491
Sinusitis	0.0533	-0.3362	-0.0582	0.1424	-0.1936	-0.1077	0.1008	-0.144	0.0141	0.1004	0.2562	0.6888
Stroke	0.1993	0.0820	0.0241	0.1575	**0.6215**	-0.1089	-0.1158	0.0518	0.1417	0.1166	0.0047	0.477
Thyroid conditions	0.0561	-0.0324	0.1969	-0.0171	-0.259	-0.1952	-0.0724	**0.4051**	-0.0085	0.0997	-0.2246	0.5725
Depression	-0.1003	-0.0794	-0.0066	**-0.8623**	-0.0063	-0.0827	-0.0707	-0.0893	0.1164	-0.0199	0.0819	0.2512
Anxiety	-0.0253	-0.0804	0.0081	**-0.8628**	-0.0677	-0.0711	-0.0445	-0.0085	0.1416	-0.0189	0.0902	0.2779
Alcohol/substance dependency	0.0250	0.0586	-0.0160	-0.2020	**0.5053**	0.0812	0.2541	-0.1209	-0.0333	0.0808	0.2539	0.4231

CKD=chronic kidney disease, COPD=chronic obstructive pulmonary disorder; CTD=connective tissue disorders; CHD=coronary heart disease; IBD=inflammatory bowel disorder; IBS=irritable bowel syndrome

11. Associations between air pollution and multimorbidity patterns (Table S8)

Table S8. Fully adjusted logistic regressions looking at associations between air pollution and multimorbidity patterns (N=364,144)

	Multimorbidity severity score	$PM_{2.5}$		PM_{10}		PM_{coarse}		NO_2	
Pattern	*M±SD*	*OR (95% CI)*	*P value*	*OR (95% CI)*	*P value*	*OR (95% CI)*	*P value*	*OR (95% CI)*	*P value*
No multimorbidity (n=207,749)	0.23±0.37	Ref		Ref		Ref		Ref	
Cardiovascular (n=2367)	3.22±1.25	1.21 (1.13 to 1.30)	**<0.001**	0.99 (0.93 to 1.06)	0.871	1.01 (0.94 to 1.08)	0.798	1.16 (1.05 to 1.27)	**0.002**
Diabetes and hypertension (n=13,846)	1.76±1.08	1.09 (1.06 to 1.13)	**<0.001**	1.03 (1.00 to 1.06)	0.052	1.03 (1.00 to 1.06)	0.072	1.10 (1.05 to 1.15)	**<0.001**
Reproductive organ (n=9563)	1.16±0.96	1.07 (1.03 to 1.11)	**<0.001**	1.01 (0.98 to 1.04)	0.668	0.98 (0.95 to 1.02)	0.399	1.05 (1.00 to 1.10)	**0.044**
Common mental health disorders (n=27,116)	1.72±0.91	1.12 (1.09 to 1.15)	**<0.001**	1.02 (1.00 to 1.04)	0.093	1.02 (1.00 to 1.04)	0.104	1.15 (1.11 to 1.18)	**<0.001**
Neurological (n=517)	3.00±1.30	1.31 (1.14 to 1.51)	**<0.001**	1.13 (0.99 to 1.30)	0.074	1.17 (1.02 to 1.35)	**0.029**	1.33 (1.11 to 1.60)	**0.002**
Gastrointestinal (n=6365)	1.68±1.14	1.19 (1.14 to 1.24)	**<0.001**	1.02 (0.98 to 1.06)	0.447	0.99 (0.94 to 1.03)	0.524	1.13 (1.07 to 1.20)	**<0.001**
Respiratory (n=2324)	2.97±1.16	1.24 (1.16 to 1.33)	**<0.001**	1.04 (0.97 to 1.11)	0.242	1.05 (0.98 to 1.13)	0.189	1.26 (1.15 to 1.38)	**<0.001**
Connective tissue, bone, and thyroid (n=1810)	1.94±1.16	1.13 (1.04 to 1.23)	**0.003**	1.03 (0.96 to 1.11)	0.410	1.01 (0.93 to 1.09)	0.877	1.10 (0.99 to 1.22)	0.078
Cancer (n=22,271)	2.41±0.80	1.04 (1.02 to 1.07)	**0.001**	1.01 (0.99 to 1.03)	0.482	1.01 (0.98 to 1.04)	0.465	1.04 (1.01 to 1.08)	**0.010**
Painful conditions (n=15,632)	1.91±0.96	1.21 (1.17 to 1.24)	**<0.001**	1.05 (1.02 to 1.08)	**<0.001**	1.02 (0.99 to 1.05)	0.199	1.11 (1.07 to 1.16)	**<0.001**
Glaucoma (n=3209)	1.60±0.99	1.00 (0.94 to 1.06)	0.977	0.99 (0.94 to 1.05)	0.817	0.99 (0.93 to 1.05)	0.685	1.04 (0.96 to 1.13)	0.353

Note: Participants with multimorbidity can belong to more than one pattern
Covariates: age, sex, ethnicity, education level, employment status, household income, smoking status, frequency of alcohol intake, BMI, physical activity, nearest major road, traffic intensity on nearest major road, greenspace (1000m), 24h noise pollution (Lden)
CI=confidence interval; OR=odds ratio; PM=particulate matter

12. Associations between air pollution and multimorbidity in participants recruited in 2010 (Table S9)

Table S9. Fully adjusted associations between air pollutants and multimorbidity in participants who completed the baseline assessment in 2010 (N=70,026).

	OR (95% CI)	*p value*
$PM_{2.5}$		
1	Ref	
2	1.06 (1.01 to 1.11)	0.012
3	1.15 (1.09 to 1.21)	<0.001
4	1.17 (1.10 to 1.24)	<0.001
$PM_{2.5\ (IQR)}$	*1.10 (1.07 to 1.14)*	*<0.001*
PM_{10}		
1	Ref	
2	1.04 (0.99 to 1.09)	0.106
3	1.08 (1.02 to 1.13)	0.004
4	1.05 (0.99 to 1.11)	0.097
$PM_{10\ (IQR)}$	*1.03 (1.01 to 1.06)*	*0.011*
PM_{coarse}		
1	Ref	
2	1.05 (1.00 to 1.10)	0.038
3	1.04 (1.00 to 1.09)	0.059
4	1.05 (0.99 to 1.11)	0.120
$PM_{coarse\ (IQR)}$	*1.00 (0.98 to 1.03)*	*0.785*
NO_2		
1	Ref	
2	1.05 (0.99 to 1.10)	0.080
3	1.03 (0.97 to 1.10)	0.295
4	1.09 (1.02 to 1.18)	0.014
$NO_{2\ (IQR)}$	*1.04 (1.01 to 1.08)*	*0.023*

Covariates: age, sex, ethnicity, education level, employment status, household income, smoking status, frequency of alcohol intake, BMI, physical activity, nearest major road, traffic intensity on nearest major road, greenspace (1000m), 24h noise pollution (Lden)
CI=confidence interval; IQR=interquartile range; OR= odds ratio; PM=particulate matter

13. Associations between air pollution and multimorbidity adjusting for assessment centre location (Table S10)

Table S10. Fully adjusted mixed effects ordinal logistic regression looking at associations between air pollution exposure and multimorbidity with a random intercept for assessment centre

	OR (95% CI)	*p value*
$PM_{2.5}$		
1	Ref	
2	1.05 (1.03 to 1.07)	0.001
3	1.08 (1.05 to 1.10)	<0.001
4	1.13 (1.10 to 1.16)	<0.001
$PM_{2.5\ (IQR)}$	*1.07 (1.06 to 1.08)*	*<0.001*
PM_{10}		
1	Ref	
2	1.01 (0.99 to 1.03)	0.235
3	1.03 (1.01 to 1.05)	0.004
4	1.04 (1.01 to 1.07)	0.004
$PM_{10\ (IQR)}$	*1.02 (1.01 to 1.03)*	*<0.001*
PM_{coarse}		
1	Ref	
2	1.03 (1.01 to 1.05)	0.002
3	1.04 (1.02 to 1.06)	<0.001
4	1.05 (1.02 to 1.08)	<0.001
$PM_{coarse\ (IQR)}$	*1.01 (1.00 to 1.02)*	*0.056*
NO_2		
1	Ref	
2	1.06 (1.03 to 1.08)	<0.001
3	1.10 (1.07 to 1.12)	<0.001
4	1.15 (1.11 to 1.19)	<0.001
$NO_{2\ (IQR)}$	*1.08 (1.07 to 1.10)*	*<0.001*

Covariates: age, sex, ethnicity, education level, employment status, household income, smoking status, frequency of alcohol intake, BMI, physical activity, nearest major road, traffic intensity on nearest major road, greenspace (1000m), 24h noise pollution (Lden), and assessment centre
CI=confidence interval; IQR=interquartile range; NO=nitrogen oxide; OR=odds ratio; PM=particulate matter

14. Associations between air pollutions and multimorbidity – two-way exposure models (Table S11)

Table S11. Associations between air pollution quartiles and multimorbidity in two-way exposure ordinal logistic regression models. Odds ratios are per increase in interquartile range (IQR) of each air pollutant (N=364,144)

	(i) Fully adjusted + $PM_{2.5}$		(ii) Fully adjusted + PM_{10}		(iii) Fully adjusted + PM_{coarse}		(iv) Fully adjusted + NO_2	
	OR (95% CI)	*p value*	*OR (95% CI)*	*p value*	*OR (95% CI)*	*p value*	*OR (95% CI)*	*p value*
NO_2								
1	Ref		Ref		Ref			
2	1.04 (1.02 to 1.06)	<0.001	1.09 (1.07 to 1.11)	<0.001	1.09 (1.07 to 1.12)	<0.001		
3	1.07 (1.04 to 1.10)	<0.001	1.14 (1.11 to 1.17)	<0.001	1.15 (1.12 to 1.17)	<0.001		
4	1.06 (1.02 to 1.10)	0.001	1.19 (1.15 to 1.23)	<0.001	1.19 (1.16 to 1.23)	<0.001		
$NO_{2\ (IQR)}$	*1.02 (1.00 to 1.04)*	*0.089*	*1.10 (1.08 to 1.11)*	*<0.001*	*1.09 (1.07 to 1.12)*	*<0.001*		
$PM_{2.5}$								
1							Ref	
2							1.07 (1.05 to 1.09)	<0.001
3							1.11 (1.08 to 1.13)	<0.001
4							1.17 (1.14 to 1.21)	<0.001
$PM_{2.5\ (IQR)}$							*1.08 (1.07 to 1.10)*	*<0.001*
PM_{10}								
1							Ref	
2							1.00 (0.98 to 1.02)	0.651
3							1.01 (0.99 to 1.03)	0.271
4							0.99 (0.96 to 1.01)	0.274
$PM_{10\ (IQR)}$							*1.00 (0.99 to 1.01)*	*0.709*
PM_{coarse}								
1							Ref	
2							1.03 (1.01 to 1.05)	0.001
3							1.02 (1.00 to 1.04)	0.017
4							1.00 (0.98 to 1.03)	0.893
$PM_{coarse\ (IQR)}$							*1.00 (0.98 to 1.01)*	*0.400*

Covariates: Age, sex, ethnicity, education level, employment status, household income, smoking status, alcohol intake frequency, BMI, physical activity, nearest major road, traffic intensity on nearest major road, greenspace (1000m), 24h noise pollution (Lden)

CI=confidence interval; IQR=interquartile range; OR=odds ratio; PM=particulate matter

15. Supplementary materials: References

1 Kephalopoulos S, Paviotti M, Anfosso-Lédée F, *et al.* Advances in the development of common noise assessment methods in Europe: The CNOSSOS-EU framework for strategic environmental noise mapping. *Sci Total Environ* 2014;**482–483**:400–10. doi:10.1016/j.scitotenv.2014.02.031

2 Generalised Land Use Database statistics for England 2005. GOV.UK. https://www.gov.uk/government/statistics/generalised-land-use-database-statistics-for-england-2005 (accessed 14 Jan 2022).
